# Novel Swelling‐Lytic Cell Death Triggered by Cargo‐Free Ionizable Lipid Nanoparticles

**DOI:** 10.1002/advs.202509208

**Published:** 2025-08-07

**Authors:** Junjun Wu, Zhennan Zhao, Hongsheng Wu, Sihuang Lin, Lin Huang, Guanjie Chen, Yi Yang, Hong Wang, Huijie Yan, Yonghui Shi, Liuyu Zhu, Guosheng Hu, Liling Zheng, Songying Ouyang

**Affiliations:** ^1^ Key Laboratory of Microbial Pathogenesis and Interventions of Fujian Province University Provincial University the Key Laboratory of Innate Immune Biology of Fujian Province Biomedical Research Center of South China College of Life Sciences Fujian Normal University Fuzhou 350117 China; ^2^ First Hospital of Quanzhou Affiliated with Fujian Medical University Quanzhou 362000 China

**Keywords:** adjuvant, cancer therapy, inflammation, lytic cell death, lipid nanoparticles

## Abstract

Lipid nanoparticles (LNPs) are recognized as robust and versatile drug delivery platforms holding significant promise for personalized and precision medicine applications. However, their intrinsic biological effects, including dose‐limiting toxicity and acute inflammation, limit their widespread application. Elucidating the underlying mechanisms paradoxically enables dual‐path optimization: targeted mitigation strategies for adverse effects and deliberate amplification of adverse effects for repurposing (“waste‐to‐resource”). Here, cargo‐free lipid nanoparticles containing the ionizable phospholipid IP9 (ipLNP) are found to induce broad swelling (bubble) morphology and lytic cell death across multiple cell types. ipLNP‐induced cell death involves reactive oxygen species (ROS) increase, lipid peroxidation, and GSDME cleavage, but is only inhibited by vitamin E among the tested inhibitors. By exploring the effects of vitamin E, lysosome‐associated lytic cell death is found to be triggered by ipLNP and mediated by lysosome membrane destabilization. Moreover, ipLNP exhibits remarkable potential as novel Th1/Th17‐directing vaccine adjuvants and emerging cancer therapeutic agents, not merely as drug carriers.

## Graphical abstract

A cargo‐free ionizable lipid nanoparticles (ipLNP) is found to induce broad swelling‐lytic cell death across multiple cell types. Cell death may be associated with lysosome membrane destabilization, involving ROS increase, lipid peroxidation, and GSDME cleavage. Moreover, ipLNP exhibits remarkable potential‌ as a novel Th1/Th17‐directing vaccine adjuvant and an emerging cancer therapeutic agent, not merely as drug carriers.

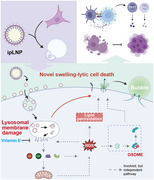

## 1 Introduction

Lipid nanoparticles (LNPs) have emerged as a revolutionary drug delivery platform.^[^ ^1^, ^2^ ^]^ The most prominent achievement lies in enabling the first FDA‐approved mRNA vaccines against COVID‐19, which have been administered billions of times globally – a testament to their clinical viability and scalability. Beyond the pandemic response, LNPs can deliver diverse payloads including siRNAs,^[^ ^3^ ^]^ CRISPR/Cas system,^[^ ^4^ ^]^ immunostimulants,^[^ ^5^ ^]^ and small‐molecule drugs.^[^ ^6^ ^]^ Moreover, their unique advantages of biocompatibility, enhanced cellular uptake, and tunable surface properties have expanded their therapeutic applications to illnesses ranging from oncological to monogenic diseases. Recent advances in “SORT” lipid strategy and protein corona design further position LNPs as a versatile, precise platform for tissue‐specific and even cell type‐specific delivery, opening a new field for LNP‐based precision medicine.^[^ ^7^, ^8^ ^]^ Continuous refinement of LNP formulations underscores their transformative role in bridging nanomedicine innovations through clinical translation.

Although LNPs have been widely studied for their delivery capabilities, their intrinsic biological effects have frequently been neglected.^[^ ^9^ ^]^ Conventional LNPs comprise ionizable lipids, helper lipids, cholesterol, and PEG‐lipids. These components, especially ionizable lipids, are directly associated with tolerability challenges, including dose‐limiting liver toxicity and other tissue injury.^[^ ^10^, ^11^, ^12^, ^13^ ^]^ Moreover, emerging evidence reveals that a number of LNPs exhibit pro‐inflammatory properties (acute inflammation), although the underlying mechanisms remain unclear.^[^ ^14^, ^15^, ^16^, ^17^ ^]^ These concerns cast a shadow over the widespread application of LNPs, particularly in the context of RNA medicine. The observed intrinsic activities of LNPs may arise from lipid interactions with immune receptors or organelle stress responses.^[^ ^9^, ^17^, ^18^ ^]^ Elucidating mechanisms involved has dual significance. First, it enables targeted strategies to mitigate the side effects by lipid formulation optimization and pathway intervention. Second, side effects could be repurposed advantageously, for instance, by leveraging inherent immunogenicity to enhance vaccine efficacy or amplifying the toxic effect for therapeutic benefit. In our previous study, an IP9‐based LNP encapsulating STING agonist was found to induce necroptosis in DC2.4 (immortalized mouse dendritic cell) and RAW‐ISG (immortalized mouse macrophage).^[^ ^5^ ^]^ Based on this, we were curious about the cytotoxicity of cargo‐free IP9‐based LNP toward different cell lines and the mechanisms involved to provide insight into LNP‐related side effects.

**Figure 1:**
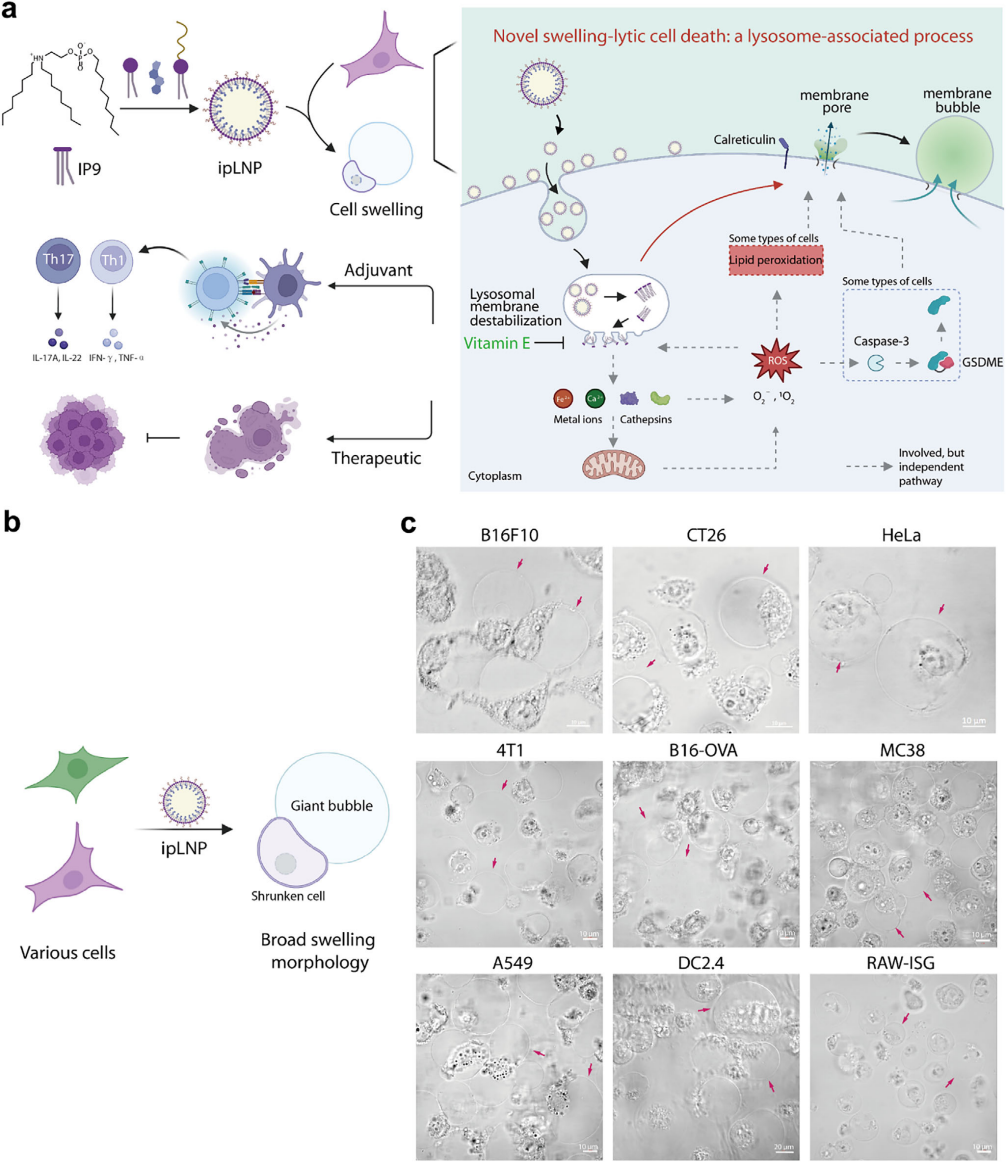
A novel swelling‐lytic cell death induced by cargo‐free ipLNP: morphology, mechanism, and potential application. a) Schematic of cargo‐free ipLNP‐induced cell death, involving morphology, mechanism, and potential application. Illustration was created in BioRender.com. b) Schematic for swelling bubble morphology induced by ipLNP. c) Representative images of swelling bubbles in various cell lines treated with ipLNP (IP9: 9–15 µg mL^−1^).

In this study, we first found that cargo‐free IP9‐based LNP (ipLNP) induces a broad bubble (swelling) morphology and lytic cell death across multiple cell types (**Figure** **1**). IP9 is a novel ionizable phospholipid identified recently.^[^ ^19^ ^]^ Given this, we anticipated that ipLNP might serve as a unique entry point for investigating the biological effects of LNPs. Following this, it was further found that lytic cell death induced by ipLNP depends on IP9 and can be blocked by osmoprotectants owing to the formation of membrane pores. Additionally, the formation of membrane pores did not result from direct extracellular membrane lysis by ipLNP. Cell death induced by ipLNP involves reactive oxygen species (ROS) increase, lipid peroxidation, and GSDME cleavage, but is only inhibited by vitamin E (VE). By investigating the inhibitory mechanism of VE, we found that ipLNP triggered a lysosome‐associated lytic cell death via lysosomal membrane destabilization. Moreover, ipLNP elicited IFN‐signatures and the activation of multiple immune pathways in vitro and in vivo. Using HR121 protein as a model antigen, ipLNP displayed robust adjuvant activity in inducing Th1/Th17 immune responses, outperforming the conventional adjuvant poly(I:C). Additionally, the broad swelling‐lytic cell death also motivated us to explore the therapeutic potential of ipLNP toward cancer. In tumor‐bearing mice, the administration of ipLNP displays certain tumor‐suppressing effects, inducing a phenotype of necrotic ulceration in tumor tissues. In summary, the ionizable lipid IP9 in ipLNP drives a lysosome‐associated lytic cell death via lysosomal membrane destabilization, which exhibits remarkable potential‌ as a novel Th1/Th17‐directing vaccine adjuvant and cancer therapeutic.

## 2 Results

### 2.1 Cargo‐Free ipLNP can Induce Broad Swelling Morphology and Lytic Cell Death Across Multiple Cell Types

The ionizable phospholipid IP9 is a pH‐switchable compound (Figure 1a) composed of ionizable tertiary amines, phosphate groups, and three lipid chains (C8, C8, and C9), which was first developed by the Siegwart group for mRNA delivery.^[^ ^19^ ^]^ In a neutral pH environment, IP9 is negatively charged due to the unprotonated tertiary amine, and cannot fuse with the membranes. While in an acidic pH environment in the endosome/lysosome, a zwitterionic head is generated in IP9 due to protonated tertiary amine, enabling IP9 insertion and interaction with membrane phospholipids (ion pairs) to form a cone shape. This facilitates subsequent hexagonal transformation of endosome/lysosome membranes, resulting in membrane integrity damage.^[^ ^19^ ^]^

To explore the cytotoxicity of cargo‐free IP9‐based LNP in different cell lines, IP9 and cargo‐free IP9‐based LNP were prepared according to the method reported by Siegwart et al.’s and our previous studies.^[^ ^5^, ^19^ ^]^ Specifically, IP9 was combined with DDAB (helper lipid), cholesterol, and DMG‐PEG2000 (the same molar ratio of 60:30:40:0.4 in Siegwart's^[^ ^19^ ^]^ and our previous work^[^ ^5^ ^]^) to obtain empty LNP, namely ipLNP, using an ethanol‐dilution procedure (Figure 1a). Upon incubating ipLNP with mouse melanoma cells B16F10, giant swelling bubbles were observed, which were obviously distinct from the normal cell morphology (Figure 1b,c). To clarify the breadth of the phenotypes induced by ipLNP, additional cell lines were tested, including mouse colon carcinoma (CT26, MC38), mouse breast tumor 4T1, B16‐OVA, human non‐small cell lung cancer A549, human cervical cancer HeLa, and human hepatoma Hep G2 (Figure 1c; Figure S1, Supporting Information). Strikingly, all cell lines above displayed a bubble morphology like that of B16F10. Besides, this phenotype was also validated in two antigen‐presenting cell (APC) lines DC2.4 and RAW‐ISG (Figure 1c). These results preliminarily indicate a universal mechanism involved in the broad bubble morphology of different cell types generated by ipLNP.

**Figure 2:**
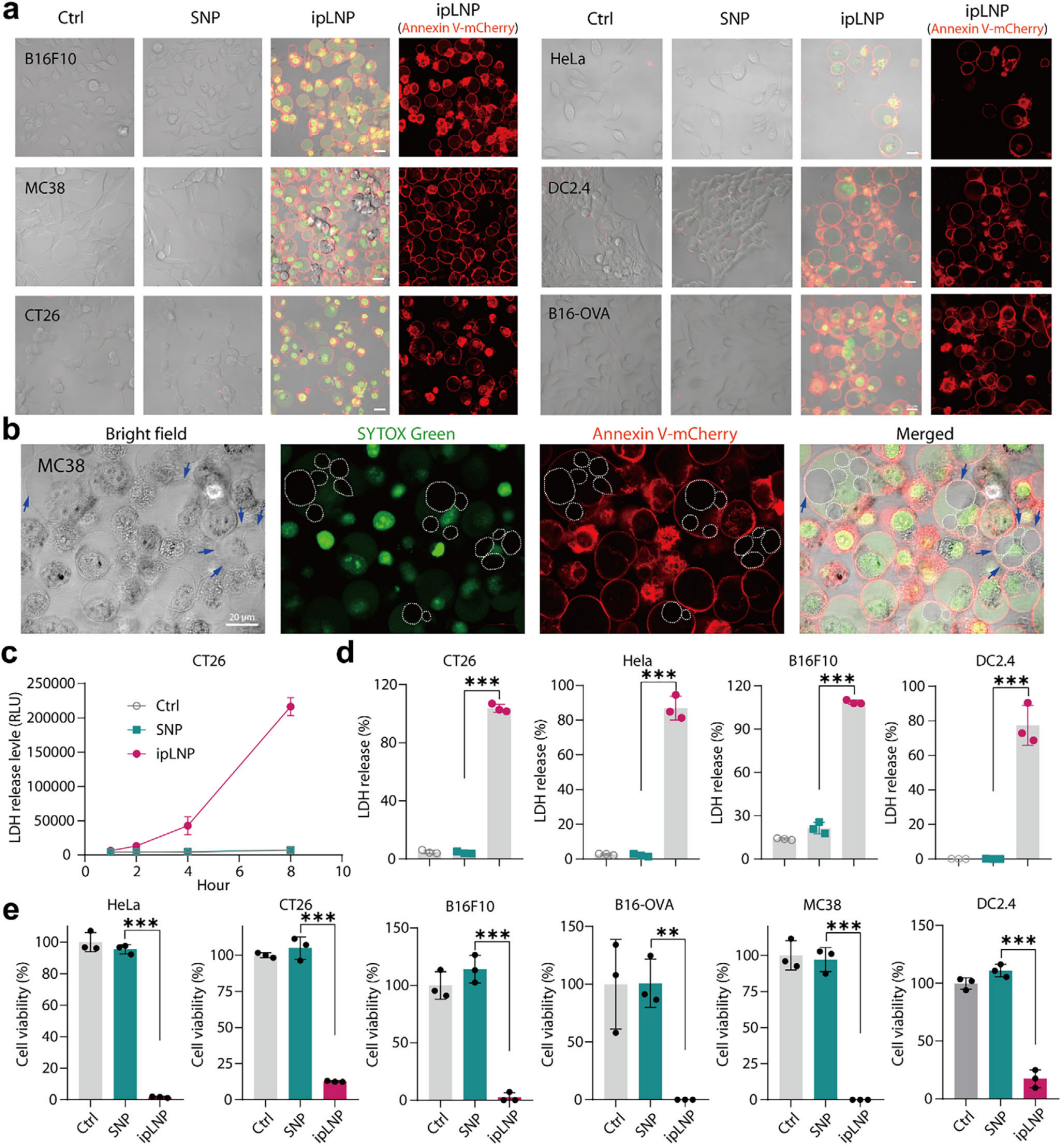
ipLNP induces cellular membrane integrity damage, contents release, and cell death. a) Confocal images of cells treated with ipLNP and stained with Annexin V‐mCherry/SYTOX Green. b) Confocal images of “bubble in bubble” in MC38 cells treated with ipLNP and stained with Annexin V‐mCherry/SYTOX Green. c) LDH release curve of CT26 cells treated with ipLNP (IP9: 9 µg mL^−1^). d) LDH release percentages in CT26, HeLa, B16F10 and DC2.4 (IP9 15 µg mL^−1^ for DC2.4, and 9 µg mL^−1^ for others). e) Cell viability of cells treated with ipLNP (IP9 15 µg mL^−1^ for DC2.4, and 9 µg mL^−1^ for others). Data were shown as mean±SD and statistical significance was analyzed with two‐tailed Student's *t*‐test, ^**^ *p*<0.01, ^***^ *p*<0.001.

To analyze the characteristics of cells with swelling bubble morphology, Annexin V‐mCherry/SYTOX Green agents were further used to stain cells above treated with ipLNP, and an FDA‐approved SM‐102‐based empty LNP (termed SNP) was used as a control (the same molar concentration as IP9). Annexin V‐mCherry can bind to phosphatidylserine on the outer surface of the abnormal membrane (a marker of apoptosis), and SYTOX Green (a cell‐impermeable dye) was used to stain the DNA of cells with compromised plasma membranes. As shown in **Figures** **2**a and S2 (Supporting Information), the cells treated with SNP (equivalent molar concentration) exhibited normal morphology and double‐negative staining, just like the blank control. However, various cells in the ipLNP group were simultaneously stained with Annexin V‐mCherry and SYTOX Green, indicating a non‐apoptotic process. Apparent arc‐like Annexin V‐mCherry signals were observed on membrane bubbles, and the nuclei of cells displayed a well‐defined profile, as evidenced by clear SYTOX Green signals (especially in confocal images of CT26, MC38), suggesting no obvious nucleus fragmentation during bubble formation. Meanwhile, weak SYTOX Green signals also appeared and were diffusely distributed in the bubble cavity (or cytosol), probably due to DNA leakage from the nuclei (Figure 2a).

In addition to these observations, “bubble in bubble” was another typical phenomenon frequently appearing in multiple cell types treated with ipLNP (Figures 1c and 2a). As illustrated in Figure 2b, the “tangent” of the membrane edges (blue arrows) between the outer and inner bubbles confirmed the inclusion relationship, and some cells contained up to six obvious inner bubbles. Furthermore, neither Annexin V‐mCherry nor SYTOX Green signal were observed in inner bubbles (dashed circles in merged image), possibly indicating their derivation from organelles without DNA. The feature of double‐positive staining in ipLNP‐treated cells was also verified by flow cytometry, showing a significantly higher staining percentage than that of SNP (Figure S3, Supporting Information).

In the late stage of ballooning, most cells displayed bubble rupture morphology (Figure 4), similar to pyroptotic cells with plasma membrane rupture (PMR).^[^ ^20^ ^]^ PMR and cellular content leakage are recognized as the critical and final steps of lytic cell death. To clarify whether ipLNP induced PMR, lactate dehydrogenase (LDH, 140 kDa) release was performed. Following ipLNP incubation, the LDH concentration in the supernatant of CT26 cells increased quickly over time, whereas the LDH concentration in SNP‐treated cells remained at the baseline level (Figure 2c, d). The robust PMR induction effect of ipLNP was also validated in HeLa, B16F10, and DC2.4 with an 80–100% LDH release percentage (Figure 2d). Consistently, ipLNP eventually caused a substantial decrease in cell viability following PMR in all six cell lines (Figure 2e).

### 2.2 Lytic Cell Death Induced by ipLNP Depends on IP9 and Can be Blocked by an Osmoprotectant Due to the Formation of Membrane Pores

**Figure 3:**
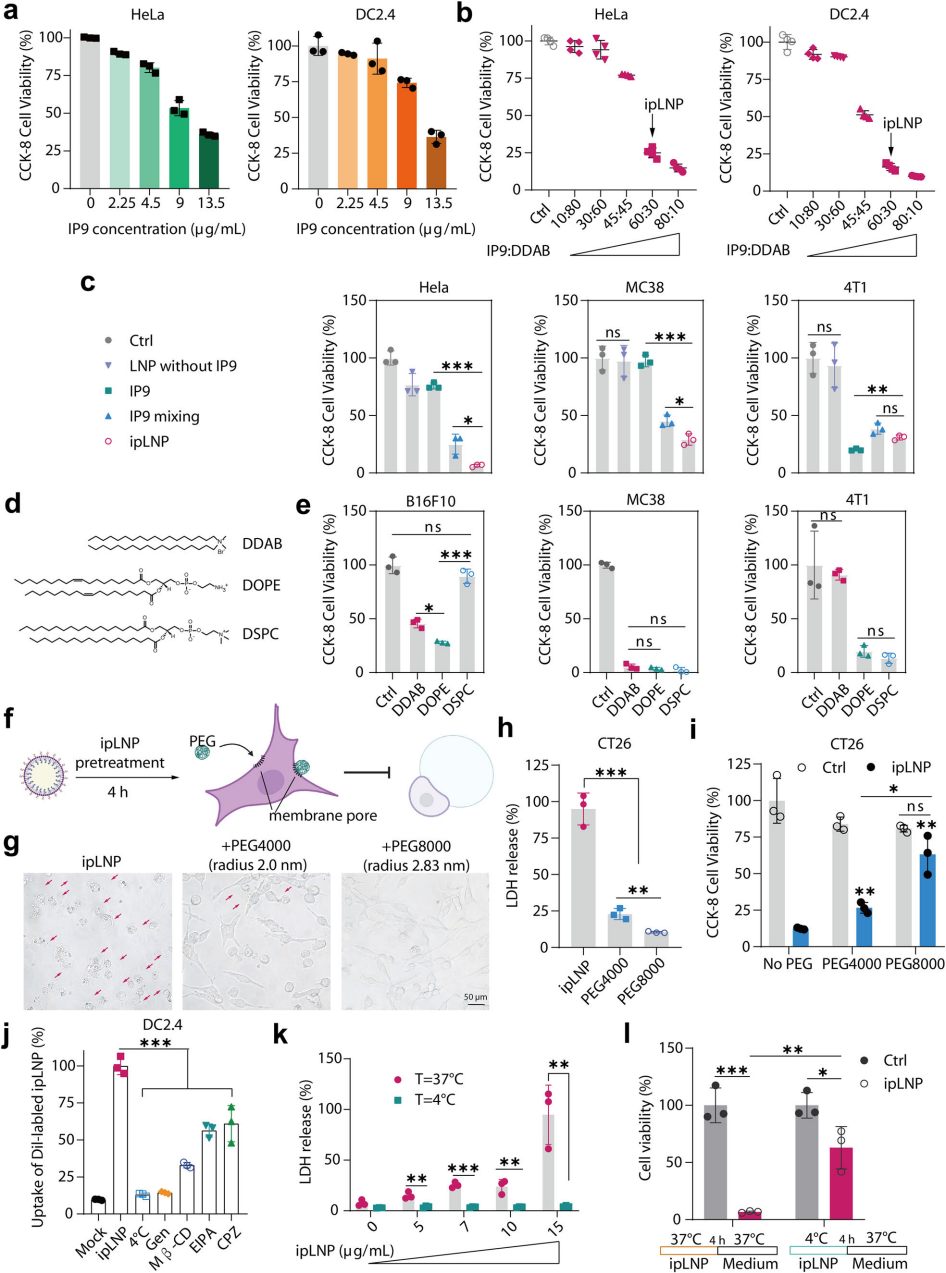
Lytic cell death induced by ipLNP depends on IP9 and can be blocked by an osmoprotectant. a) Cell viability of HeLa and DC2.4 cells treated with different concentrations of ipLNP. b) Cell viability of HeLa and DC2.4 cells treated with ipLNPs prepared from different molar ratios of IP9: DDAB (cholesterol: 4.7 µg mL^−1^). c) Cell viability of HeLa, MC38 and 4T1 cells treated with different formulations of IP9 and LNP without IP9 (IP9: 6 µg mL^−1^). IP9 group, direct addition of IP9 into cells. IP9 mixing group, preparation with ethanol‐dilution procedure through quickly mixing IP9 ethanol solution and citric acid/sodium citrate buffer. d,e) Cell viability of B16F10, MC38, and 4T1 cells treated with IP9‐based LNP using different helper lipids (IP9: 6 µg mL^−1^) for a short incubation time of 9–11 h. f) Schematic of the membrane pore blocking effect of osmoprotectant PEG on ipLNP‐pretreated cells. g–i) Images (g), LDH release (h), and cell viability (i) of ipLNP‐pretreated CT26 cells (IP9: 5 µg mL^−1^) after incubation of PEG4000 and PEG8000 (0.1 g mL^−1^). j) Cellular uptake mechanism analysis of ipLNP using inhibitors and low temperature. k) LDH release of ipLNP‐treated CT26 cells at incubation temperatures of 4 °C and 37 °C. l) Cell viability of CT26 cells treated with process of 4/37 °C ipLNP incubation (IP9: 7 µg mL^−1^) for 4 h‐washing‐37 °C medium incubation. Data were shown as mean±SD and statistical significance was analyzed with two‐tailed Student's *t*‐test and one‐way ANOVA with Tukey test, ns no significance, ^*^ *p*<0.05, ^**^ *p*<0.01, ^***^ *p*<0.001.

Based on the unique bubble morphology described above, factors affecting the properties of ipLNP were further explored. As illustrated in **Figure** **3**a, ipLNP displayed concentration‐dependent cytotoxicity. At the same total lipid concentration, cytotoxicity also increased with increasing molar ratios of IP9 and helper lipid DDAB, indicating a critical role of IP9 (Figure 3b). To verify that, controls including LNPs without IP9, IP9 alone (directly dissolved formulation), and IP9 mixing (prepared with an ethanol‐dilution procedure), were adopted. LNP without IP9 almost had no impact on cell viability, and IP9 alone possessed unstable cell death‐inducing capability in different cell lines (Figure 3c). In contrast, IP9 mixing and ipLNP both generated a robust cell viability decrease, and ipLNP exhibited a more prominent lethal effect than that of IP9 mixing. These results firmly demonstrated that IP9 was indispensable for ipLNP‐induced lytic cell death, and the nanoformulation contributed remarkably to the cell‐death inducing activity of IP9.

Helper lipids are another key component, second only to ionizable lipids in influencing the performance of LNP. Given that, IP9 nanoformulations containing different helper lipids, including cationic lipid DDAB, zwitterionic lipid DOPE, and neutral lipid DSPC, were prepared to evaluate the roles in IP9‐mediated lytic cell death (Figure 3d). After a short incubation time (9‐11 h), three helper lipids‐containing LNPs induced different cell death rates toward B16F10 (DOPE>DDAB>DSPC), MC38 (DOPE = DDAB = DSPC) and 4T1 (DOPE = DSPC>DDAB) cells (Figure 3e). The impact of helper lipids on the cytotoxicity of IP9‐based LNPs were also observed in CT26 and DC2.4 cells (Figure S4, Supporting Information). However, the trends in surface charge and cellular uptake levels were not matched with the cytotoxicity trends of IP9‐based LNPs in CT26 and DC2.4 cells (Figure S4, Supporting Information), which needed further investigation to reveal the special mechanism involved.

Generally, cell swelling/ballooning in pyroptosis and necroptosis is mediated by a common feature, namely, pore formation in the plasma membrane.^[^ ^20^, ^21^ ^]^ The pore‐induced damage to membrane integrity triggers a net influx of water and an increase in osmotic pressure, eventually resulting in cell collapse (PMR) mediated by NINJ1 activation and other factors.^[^ ^22^ ^]^ To explore whether pore formation was involved in ipLNP‐induced ballooning and cell death, polyethylene glycols (PEG) of different sizes, a commonly used osmoprotectant,^[^ ^23^, ^24^ ^]^ were adopted to block the pores with size effect (Figure 3f). Besides, PEG was added after the cells had already been treated with ipLNP for 4 h (sufficient for cellular uptake), to avoid the direct impact of PEG on ipLNP. Compared with ipLNP alone, PEG coincubation significantly diminished the formation of cell bubbles and retained the normal morphology in a size‐dependent manner (Figure 3g). Particularly, in the presence of the higher‐molecular‐weight PEG8000 (hydrated radius 2.7 nm), an almost complete phenotype inhibition was observed. PEG8000 addition substantially reduced LDH release and increased cell viability (nearly that of PEG8000 alone) (Figure 3h,i). These results proved that membrane pores were indeed generated during ipLNP incubation. And based on the moderate inhibitory effect of PEG4000 (hydrated radius 1.8 nm), the pore diameter was estimated to be 3.6‐5.4 nm, similar to that of pyroptosis and ferroptosis.^[^ ^23^, ^24^ ^]^

The next question that arises is: how are the pores formed. We speculated that the triggering factors may originate from two aspects: intracellular pathways‐induced pores (such as pyroptosis, necroptosis), or direct extracellular membrane lysis (such as an oncolytic peptide). To clarify that, the uptake mechanism was studied using DiI‐labeled ipLNP and multiple inhibitors. The results showed energy‐dependent, caveolin and lipid raft‐mediated endocytosis (Figure 3j; Figure S5, Supporting Information). Distinct from the high LDH release level in normal condition (37 °C), ipLNP did not induce cellular LDH release in 4 °C incubations, even at a concentration up to 15 µg/mL (Figure 3k; Figure S6, Supporting Information). Accordingly, ipLNP displayed a weaker cell death induction activity after a process of 4 °C incubation (4 h)‐washing‐37 °C medium incubation, than that after a process of 37 °C incubation (4 h)‐washing‐37 °C medium incubation (Figure 3l). These indicated a need for cell uptake of ipLNP to induce lytic cell death.

**Figure 4:**
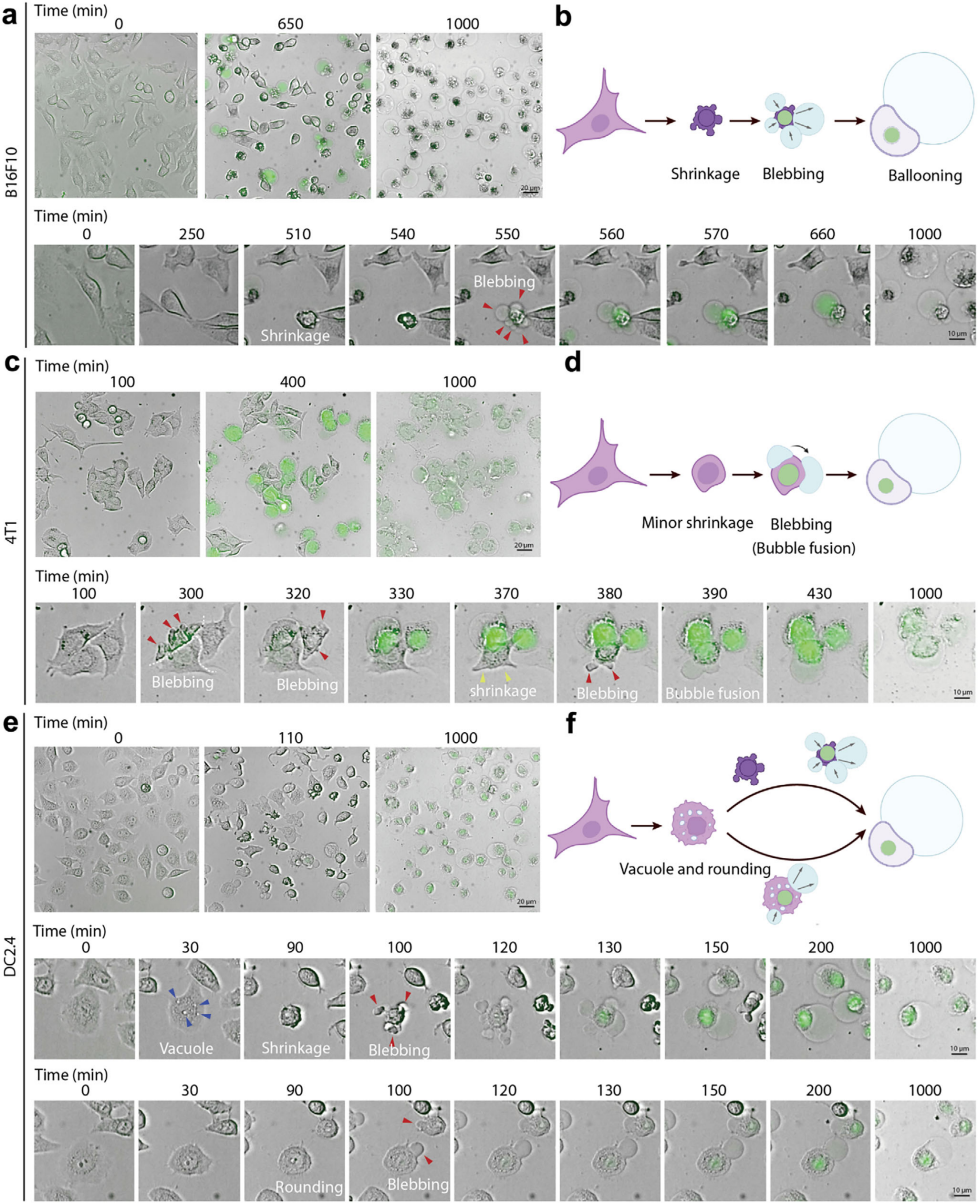
Cell death triggered by ipLNP undergoes a shrinkage/rounding‐blebbing‐ballooning transition and is not mediated by direct extracellular membrane lysis. a–f) Time‐lapse images and schematic for the morphological change process of B16F10 cells (a,b), 4T1 cells (c,d), and DC2.4 cells (e,f) treated with ipLNP (IP9: 5 µg mL^−1^) for ≈24 h, and SYTOX Green was added to analyze the cellular membrane integrity.

To further exclude the mechanism of direct extracellular membrane lysis, a time‐lapse high‐content analysis was performed using B16F10, DC2.4, and 4T1 cells (**Figure** **4**; Videos S1–S3, Supporting Information). Generally, the progression of the phenotype associated with direct extracellular membrane lysis is much more rapid (≈15 min^[^ ^25^, ^26^ ^]^) than that of intracellular pathways (>1 h). Three cell lines exhibited distinct and long peak times of swelling phenotype (B16F10: 10.8 h, DC2.4: 1.8 h, 4T1: 6.7 h) (Figure 4), suggesting that direct extracellular membrane lysis might not account for the ipLNP‐induced membrane pores. Besides, different ballooning processes were also observed. In B16F10, ipLNP incubation resulted in a unique shrinkage‐blebbing‐ballooning transition (Figure 4a,b; Video S1, Supporting Information). In 4T1, the process involved minor shrinkage‐blebbing‐ballooning transition, like that of B16F10 but with minor changes in cell volume (Figure 4c,d; Video S2, Supporting Information). Interestingly, two different morphological changes occurred simultaneously in DC2.4, including vacuole‐shrinkage‐blebbing‐ballooning and vacuole‐rounding‐blebbing‐ballooning (Figure 4e,f; Video S3, Supporting Information). No SYTOX Green signals were observed in any cell lines during cell shrinkage or rounding, indicating the membrane integrity was maintained during cell deformation. Notably, during the morphological transformation, cells gradually detached from the bottom of the dish and floated in the culture medium with a giant bubble. This seemed distinct from the cells undergoing pyroptosis, or extracellular membrane lysis, which generally remained stable at the bottom. Collectively, three cell lines treated with ipLNP underwent a similar and relatively slow process of ballooning, probably driven by intracellular pathways.

### 2.3 ipLNP‐Induced Cell Death Involves ROS Increase, Lipid Peroxidation, and GSDME Cleavage, but is Only Inhibited by Vitamin E

**Figure 5:**
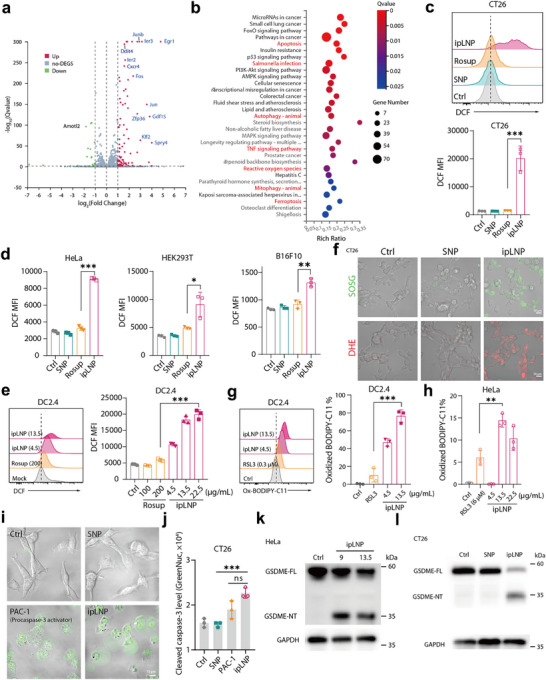
ipLNP‐induced cell death involves ROS increase, lipid peroxidation, and GSDME cleavage. a, b) Transcriptomic analysis of HeLa cells treated with ipLNP and the results were shown as a volcano plot (a) and KEGG pathway of the differentially expressed genes (DEGs) (b). c–e) ROS level in cells treated with ipLNP (IP9 8 µg mL^−1^ for CT26, and 13.5 µg mL^−1^ for others), detected with DCFH‐DA probe. Rosup was used as a positive control. f, ROS type analysis of CT26 cells using SOSG and DHE probe (IP9: 9 µg mL^−1^). g, h) Lipid peroxidation analysis of DC2.4 (g) and HeLa (g) cells using BODIPY‐C11 probe. RSL3 was used as a positive control. i, j) Confocal images and fluorescence intensity of ipLNP‐treated CT26 cells using GreenNuc caspase‐3 probe. PAC‐1 (100 µm) was used as a positive control. k, l) Western blot analysis of cleaved GSDME in HeLa and CT26 cells treated with ipLNP. Data were shown as mean±SD and statistical significance was analyzed with two‐tailed Student's *t*‐test, ns no significance, ^*^ *p*<0.05, ^**^ *p*<0.01, ^***^ *p*<0.001.

To explore the intracellular mechanism involved in ipLNP‐induced lytic cell death, HeLa cells treated with ipLNP were subjected to transcriptomic analysis. And a series of cell stress‐related genes were significantly upregulated in ipLNP‐treated cells compared with control, including *Ier3, Ier2, Fos, and Gdf15* (**Figure** **5**a). The Kyoto Encyclopedia of Genes and Genomes (KEGG) analysis further indicated the involvement of pathways like ROS, ferroptosis, and apoptosis (Figure 5b). Based on transcriptomic analysis, intracellular ROS levels were first evaluated by DCFH‐DA probe, because of its critical role in cellular homeostasis. Indeed, ipLNP incubation induced ROS level elevation in multiple cell lines (tumor cells and dendritic cells) in a concentration‐dependent manner, and ipLNP‐treated CT26 cells displayed the maximum increase (Figure 5c–e). And ROS type contained singlet oxygen (^1^O_2_, detected by SOSG probe) and superoxide radicals (O_2_^−^, detected by DHE probe) (Figure 5f). Recently, several studies have clarified the central role of ROS in ferroptosis through promoting lipid peroxidation,^[^ ^27^ ^]^ and pyroptosis through facilitating caspase activation and gasdermin cleavage/post‐translational modification.^[^ ^28^ ^]^ In accordance with transcriptomic analysis, ipLNP generated the lipid peroxidation in tested cell lines to different degrees (DC2.4 80%, HeLa 15% and CT26 1.6%) in a concentration‐dependent manner (Figure 5g,h; Figure S7, Supporting Information), probably due to variations in cellular sensitivity to ferroptosis. Additionally, increased cellular ROS generally leads to caspase activation, particularly caspase‐3. To explore whether caspase‐3 was activated, a cell‐permeable GreenNuc™ caspase‐3 substrate was adopted which can release coupled DNA dye after specific cleavage by activated caspase‐3. Similar to cells treated with the positive control PAC‐1 (a procaspase‐3 activator), obvious fluorescence intensity was also observed in the ipLNP group, but not in the SNP group (Figure 5i,j). Meanwhile, the cells in PAC‐1 and ipLNP presented a similar cell swelling morphology, suggesting the existence of a caspase‐3/gasdermin E (GSDME) axis. To investigate this further, cells treated with ipLNP were subjected to western blot analysis and ipLNP indeed triggered GSDME cleavage in CT26 and HeLa cells (with relatively high GSDME expression levels) (Figure 5k,l; Figure S8, Supporting Information).^[^ ^29^ ^]^ In summary, these findings revealed that ROS, lipid peroxidation, and GSDME cleavage were involved in ipLNP‐induced cell death.

**Figure 6:**
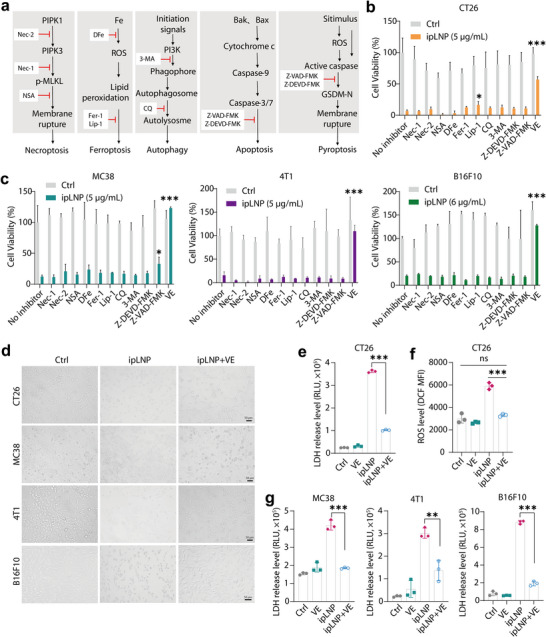
Comprehensive inhibitor screening against typical cell death pathways and only vitamin E exhibits a rescue effect. a) Schematic for cell death pathways and targeting inhibitors. b,c) Viability of cells treated with ipLNPs and inhibitors (n = 3). Inhibitors were preincubated with cells for 12 h, and then media were replaced with fresh inhibitors and ipLNP, and incubated for 24 h. d–g) Cell images (d), LDH level (e,g) and ROS level (f) of cells treated with ipLNP and VE (75 µm). Data were shown as mean±SD and statistical significance was analyzed with two‐tailed Student's *t*‐test, ns no significance, ^*^ *p*<0.05, ^**^ *p*<0.01, ^***^ *p*<0.001.

To further elucidate the contribution of ROS, lipid peroxidation, and pyroptosis in ipLNP‐induced cell death, a comprehensive screening was conducted, using a series of inhibitors of typical cell death pathways including necroptosis, ferroptosis, autophagy, apoptosis, and pyroptosis (**Figure** **6**a). Among them, vitamin E (VE) was initially adopted against cellular ROS and lipid peroxidation, owing to its lipophilicity and antioxidant activity. Unexpectedly, in CT26, nearly all inhibitors, except lip‐1 and VE, exhibited no obvious impact on rescuing cell death induced by ipLNP (Figure 6b), revealing a caspase‐independent process. Besides, compared with lip‐1, VE generated a higher cell viability (≈60%), probably showing a dispensable role of lipid peroxidation. To further validate the generalizability of observations, more cell lines were studied. Similarly, VE incubation remarkably suppressed the cytotoxicity of ipLNP in MC38, 4T1 and B16F10 (Figure 6c). And other inhibitors still did not show any alleviation effects, except for the pan‐caspase inhibitor Z‐VAD‐FMK in MC38. The inhibitory effect of VE toward ipLNP was further confirmed by the characterization of cell morphology (Figure 6d; Figure S9, Supporting Information), LDH release (Figure 6e,g) and ROS level (Figure 6f). The normal cell morphology was observed in multiple cell lines treated with ipLNP+VE, along with the preservation of cell membrane integrity and cellular ROS balance. Overall, these findings preliminarily indicated a unique mechanism involved in ipLNP‐induced cell death, which can be specifically blocked by VE.

### 2.4 ipLNP Induces a Lysosome‐Associated Lytic Cell Death

**Figure 7:**
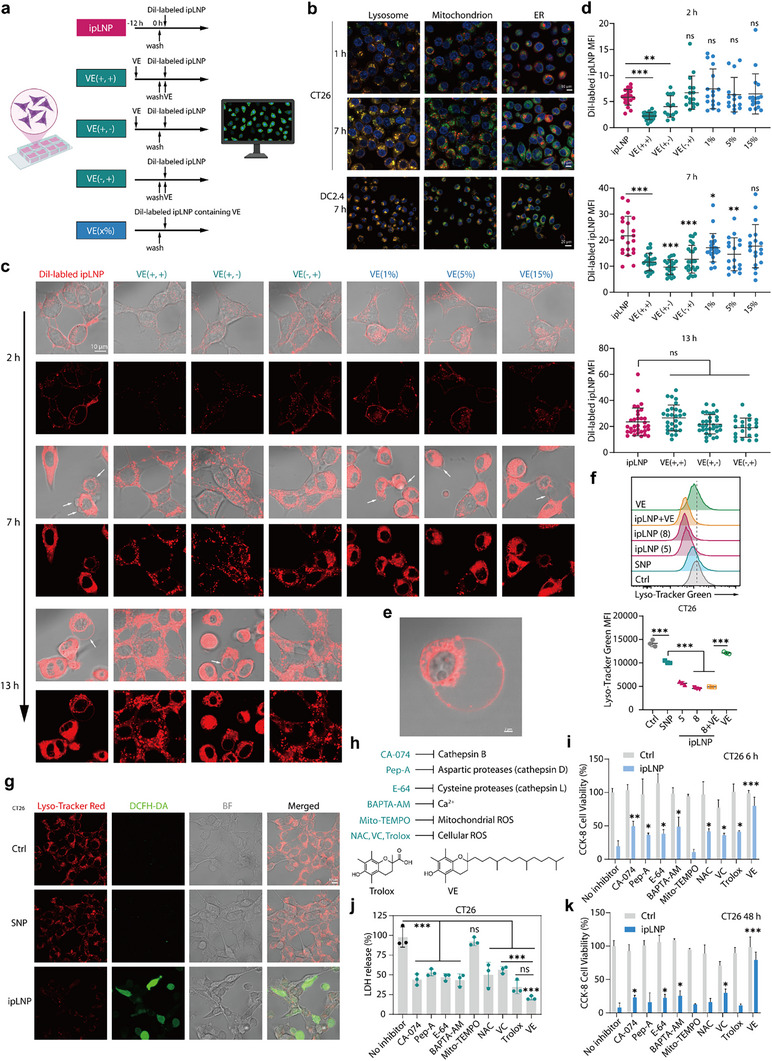
ipLNP induces a lysosome‐associated lytic cell death via lysosomal membrane destabilization. a) Schematic of DiI‐labeled ipLNP and VE‐containing incubation groups design. b) Organelle co‐localization analysis of DiI‐labeled ipLNP in CT26 and CD2.4 cells. Lysosome was stained with Lyso‐Tracker Green. Mitochondria were stained with Mito‐Tracker Green. ER was stained with ER‐Tracker Green. c–e) A time‐gradient confocal microscopy imaging (c,e) and quantified intensity (d) of CT26 cells treated with indicated DiI‐labeled groups. f) Flow cytometry analysis of lysosome acidification using Lyso‐Tracker Green as probe after ipLNP incubation for 6 h. g) Confocal microscopy imaging of lysosome acidification (Lyso‐Tracker Red) and ROS (DCFH‐DA). h) Cathepsin inhibitors, ion chelators, and ROS scavengers used for cell death mechanism analysis. i–k) Cell viability (i, 6 h, k, 48 h) and LDH release (j) of CT26 cells treated with indicated inhibitors (n = 3). Data were shown as mean±SD and statistical significance was analyzed with two‐tailed Student's *t*‐test, ns no significance, ^*^ *p*<0.05, ^**^ *p*<0.01, ^***^ *p*<0.001.

Considering the specific and robust activity in inhibiting ipLNP‐induced lytic cell death, VE may serve as an effective starting point for investigating the mechanism of ipLNP. To do that, ipLNP was labeled with DiI for analyzing the intracellular distribution (**Figure** **7**a). Consistent with the finding of energy‐dependent endocytosis in Figure 3j, clear dot‐like colocalization of ipLNP with lysosomes, but not mitochondria and endoplasmic reticulum (ER), was observed in CT26 and DC2.4 (Figure 7b). To further elucidate the impact of VE on ipLNP activity, a set of VE incubation procedures was designed, including VE(+,+), VE(+,‐), and VE (‐,+) (Figure 7a). Besides, VE was also co‐assembled into DiI‐labeled ipLNP with a molar ratio of 1%‐15% to explore whether VE in ipLNP displayed suppression effects. Based on that, a time‐gradient confocal microscopy imaging was performed (Figure 7c–e).

At an incubation time of 2 h, obvious DiI signals were mainly observed on the surface (curve‐like) and cytoplasm (dot‐like) of cells treated with ipLNP alone (Figure 7c), indicating a cellular endocytosis process. Cells incubated with VE(‐,+), VE(1%‐15%) presented a highly similar situation. Distinctly, fluorescent signals in VE(+,+) and VE(+,‐) were apparently weaker than those of ipLNP alone. The trend was also evidenced by the quantified fluorescent intensity of the 2 h cell (Figure 7d). These indicated that VE pretreatment reduced the cellular uptake of ipLNP in the early stage, and VE did not directly influence ipLNP to inhibit uptake. At an incubation time of 7 h, the prominent cell rounding and blebbing (white arrow) emerged in cells treated with ipLNP and VE(1%‐15%), indicating an early stage of lytic cell death. Besides, the fluorescent signals displayed a model of diffuse distribution in the cytoplasm, especially in rounded cells. Cells incubated with VE(+,+), VE(+,‐), and VE (‐,+) still retained their normal morphology, along with dot‐like fluorescent signals in the cytoplasm. The quantified fluorescence intensity of 7 h cells further revealed a significantly lower uptake of ipLNP in cells with VE(+,+), VE(+,‐), and VE (‐,+), compared with cells incubated with ipLNP alone. At an incubation time of 13 h, VE(+,‐) treated cells displayed a phenotype of cell rounding and blebbing, which was almost identical to that of cells treated with ipLNP alone. And the cells of VE(+,+) and VE (‐,+) maintained a normal state, with an increased fluorescence intensity relative to that of 7 h (Figure 7c,d).

Indeed, the quantified data showed that the cells of four groups were at the same level in terms of ipLNP uptake and might have reached a plateau after 13 h incubation (Figure 7d). Notably, integrating images and data from time points of 7 and 13 h, it could be found that normal cell morphology was tightly correlated with the dot‐like distribution of DiI‐labeled ipLNP (relatively intact lysosomes), and cell rounding was closely linked to diffuse distribution (lysosomal membrane destabilization). The results might reveal a protective mechanism of VE against ipLNP through stabilization of the lysosomal membrane. This was consistent with the lysosomal membrane‐destabilizing properties of IP9.^[^ ^19^ ^]^ Meanwhile, apparent DiI signals were observed on bubbles of ipLNP‐treated cells (Figure 7e), probably because of membrane component exchange or direct interaction between ipLNP and the cellular membrane.

Changes in acidification are generally associated with lysosome damage.^[^ ^30^ ^]^ In line with expectation, ipLNP incubation (for 6 h) remarkably induced lysosomal deacidification, as evidenced by the reduced fluorescence intensity of Lyso‐Tracker Green (Figure 7f). SNP also induced lysosomal deacidification with a smaller magnitude, indicating greater damage to the lysosome caused by ipLNP (IP9) than caused by SNP (SM‐102). Of note, VE treatment was unable to rescue lysosomal deacidification at an early stage, possibly indicating that its inhibitory effect on ipLNP did not involve the rescue of lysosomal acidification. Lysosomal deacidification was further verified by confocal imaging, along with increased ROS production (Figure 7g). The increased ROS levels observed in multiple cell lines might exacerbate the destabilization of lysosomal membranes via lipid peroxidation, as evidenced in Figure 5g,h.

In general, the damage to lysosomal membrane integrity leads to the content leakage into the cytoplasm, including cathepsins (cathepsin B, D, and L) and metal ions (Fe^2+^, Zn^2+^), which may trigger apoptotic or nonapoptotic cell death pathways.^[^ ^31^ ^]^ To elucidate the mechanisms involved in subsequent cell death after lysosome damage, a range of inhibitors were adopted (Figure 7h), including cathepsin inhibitors, Ca^2+^ chelator, and ROS scavengers. Among them, Trolox, a hydrophilic analogue of VE with potent antioxidant effect, was used as a control of VE. After a short incubation time of 6 h, cathepsin inhibitors (CA‐074, Pep‐A, and E‐64), Ca^2+^ chelator, and cellular ROS scavengers (NAC, VC, and Trolox) displayed a delayed effect of ipLNP cytotoxicity (Figure 7i)‌. However, the addition of mitochondrial ROS scavenger Mito‐TEMPO further accelerated cell death. This result was further confirmed using the LDH release assay (Figure 7j). After a long incubation time of 48 h, a relatively weak inhibitory effect on cell death was observed in groups of CA‐074, E‐64, BAPTA‐AM, and VC (Figure 7k). This might indicate a lysosomal contents‐involved, but not dependent, cell death process. Indeed, the trend of weak or no rescue effect was observed in 4T1, B16F10, and MC38 treated with the cathepsin inhibitors combination (CA‐074+Pep‐A+E‐64) and metal chelators combination (DFe+TPEN+BAPTA‐AM) (Figure S10, Supporting Information). In summary, ipLNP induced a lysosome‐associated lytic cell death through lysosomal membrane destabilization (or lysosomal membrane permeabilization). Besides, stabilization of the lysosomal membrane could be the dominant inhibition mechanism of VE, and ROS consumption might be an ancillary effect, as evidenced by the ineffectiveness of Trolox.

### 2.5 ipLNP Elicits IFN‐Signatures and Multiple Immune Pathways Activation In Vitro and Vivo

**Figure 8:**
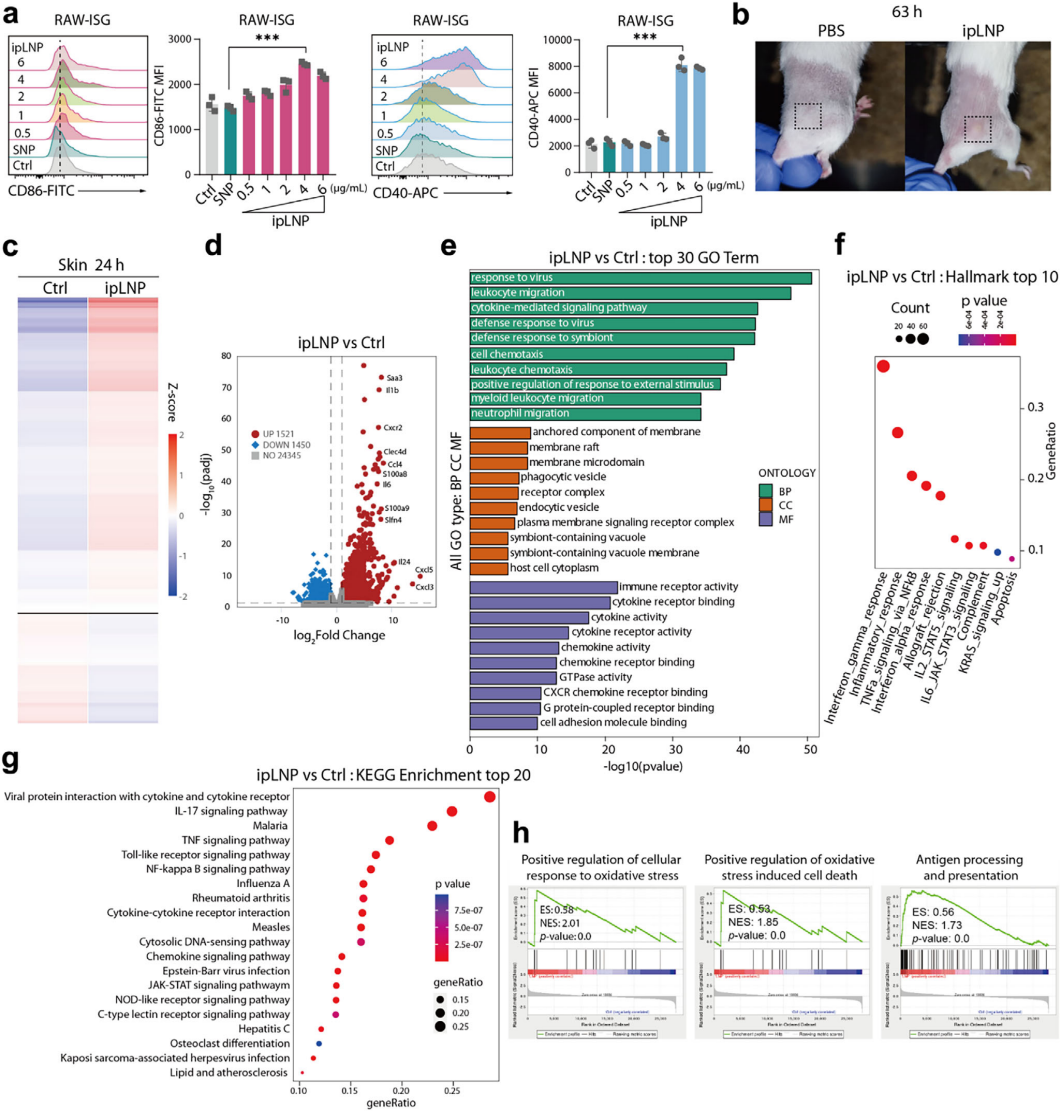
Immunostimulation and inflammation effect of ipLNP in vitro and in vivo. a) Co‐stimulatory molecules CD40 and CD86 expression on RAW‐ISG cells after incubation with low concentrations of ipLNP. b) Images of abscess and lesion on the skin of mice intradermally injected with ipLNP (IP9 dose 90 µg). c) Heatmap of DEGs in skins of indicated groups. d) Volcano plot of ipLNP versus Ctrl. e–g) GO, Hallmark, and KEEG analysis of cluster of DEGs in ipLNP versus Ctrl. h) Oxidative stress, oxidative stress induced cell death, and antigen‐presenting pathways in GSEA. Data were shown as mean±SD, and statistical significance was analyzed with two‐tailed Student's *t*‐test, ^***^ *p*<0.001.

As depicted in Figure 3a, ipLNP induced cell death was a concentration‐dependent process in both HeLa and immune‐related DC2.4 cells. Furthermore, the elevation of ROS, which has been shown to be associated with immune activation and inflammatory responses,^[^ ^32^ ^]^ was commonly observed across various cell types following treatment with ipLNP. This motivated us to further explore the immune properties of ipLNP at low concentrations in vitro. Using RAW‐ISG as a model cell line, ipLNP remarkably upregulated the expression of the co‐stimulatory molecules CD40 and CD86 in a concentration‐dependent manner (**Figure** **8**a). At a 4 µg mL^−1^ of IP9, ipLNP reached the balance point between macrophage maturation and cell death to elicit the optimal co‐stimulatory molecules expression. This result demonstrated the dual role of ipLNP: immune activation at low concentrations and cell death induction at high concentrations. Besides, the immune activation of ipLNP might be derived from the moderate destruction of the lysosome membrane.

To investigate the observation in vivo, ipLNP was intradermally injected into the right flank of mice at a dose of 90 µg IP9. Accordingly, obvious skin abscesses and lesions were observed in vivo after an intradermal injection of ipLNP (Figure 8b). And a local retention effect was observed at the injection site after the intradermal administration of DiI‐labeled ipLNP and imaging with IVIS Spectrum (Figure S11, Supporting Information). To further clarify the mechanisms involved, skin samples from sites were collected for transcriptomic analysis 24 h after administration. The heatmap of DEGs displayed an obviously different profile between ipLNP and control (Figure 8c). Besides, a range of immune function‐related genes were significantly upregulated in ipLNP‐treated mice skins compared with that of naïve mice, including proinflammatory cytokines (*Il1b, Il6, Il24*), chemokines (*Ccl4, Cxcl3, Cxcl5*), type I interferon (*Irf7*), DAMPs (*S100a8, S100a9*), and immune receptors (*Clec4d, Clec4e, Cxcr2*) (Figure 8d). Gene Ontology (GO) enrichment analysis further clarified that ipLNP‐induced responses in the skin were mainly associated with the host immune defenses in Biological Process, and cytokine and chemokine activities in Molecular Function (Figure 8e), indicating an immunostimulatory property. In addition, the DEGs induced by ipLNP were primarily involved in the Cellular Component of membrane structures (anchored component, raft, microdomain), phagocytic vesicle, endocytic vesicle, symbiont‐containing vacuole, and host cell cytoplasm (Figure 8e). This closely matched the internalization process of ipLNP observed in vitro within DC2.4 and CT26, namely energy‐dependent endocytosis mechanism. Meanwhile, the results also suggested that the interaction between the vesicle/vacuole membrane and the contained ipLNP led to the skin abscesses and lesions, consistent with lysosome accumulation and cell death in vitro. The Hallmark and KEGG analysis of the cluster of DEGs upregulated by ipLNP presented an enrichment for type I, II interferon and pro‐inflammatory responses (Figure 8f,g), which might be driven by IL‐17 signaling and multiple pattern recognition receptor (PRR) pathways, including toll‐like receptor (TLR), cytosolic DNA‐sensing (cGAS‐STING), NOD‐like receptor (NLR), and C‐type lectin receptor (CLR). What's more, cellular response to oxidative stress, oxidative stress‐induced cell death, and antigen‐presenting pathways were also observed in the gene set enrichment analysis (GSEA) (Figure 8h), consistent with increased ROS phenotype in various cell lines in vitro. Collectively, the co‐stimulatory molecules expression in vitro and transcriptomic analysis in vivo revealed that ipLNP induced a potent innate immune activation and proinflammatory effect, possibly mediated by the damage of lysosomal membrane integrity in cells at injection sites.

### 2.6 ipLNP Possesses Robust Adjuvant Activity in Inducing Th1/Th17 Immune Responses

**Figure 9:**
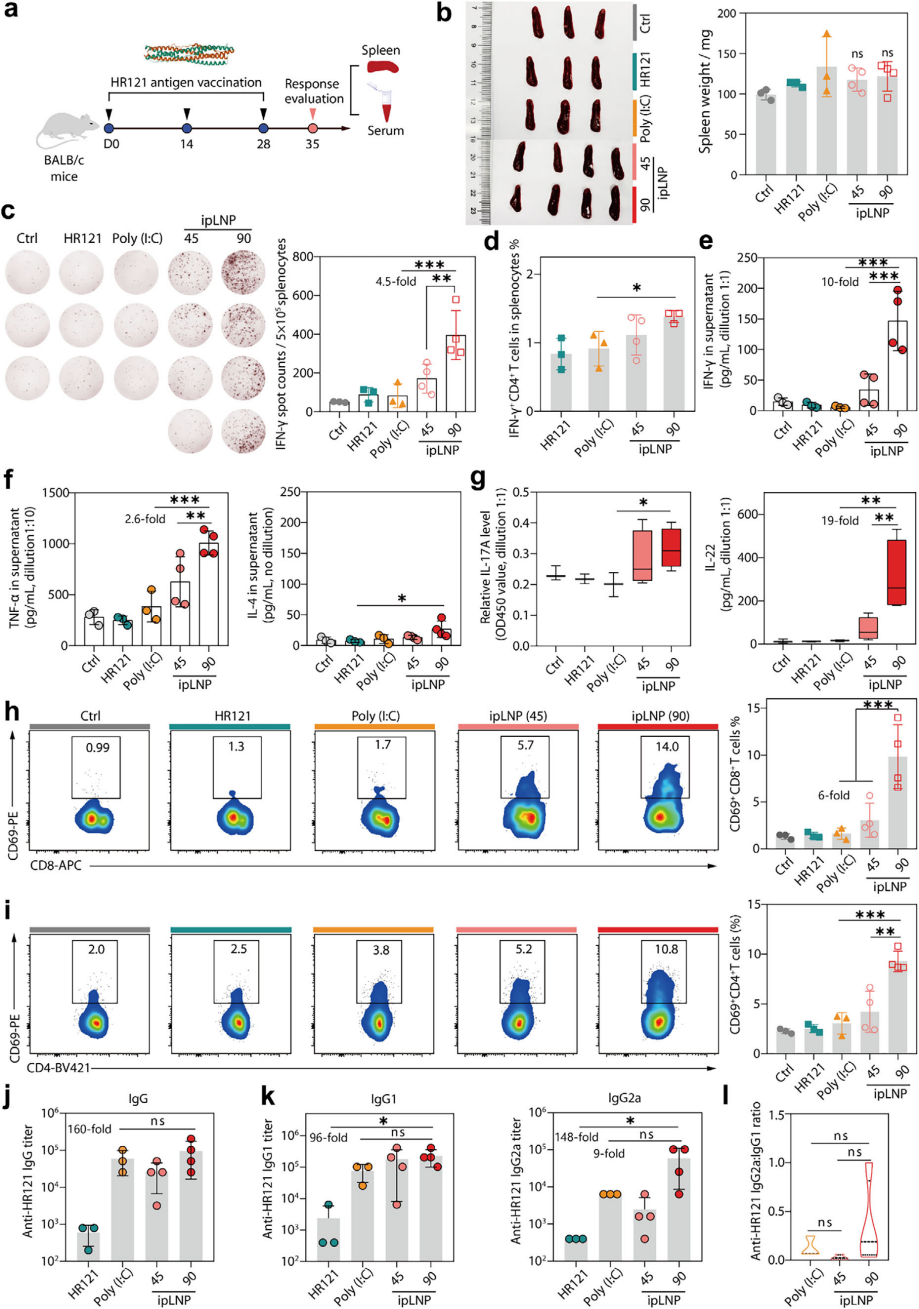
ipLNP possesses robust adjuvant activity toward inducing Th1 and Th17 immunity, outperforming the conventional adjuvant poly(I:C). a) Vaccination and sample harvest schematic for BALB/c mice intramuscularly injected with vaccine formulations (IP9 dose: 45/90 µg, poly (I:C) dose: 50 µg, HR121 dose: 10 µg). b) Images and weight of spleens from immunized mice. c) IFN‐γ ELISpot images and corresponding spot quantification of splenocytes after restimulating with 50 µg mL^−1^ HR121 for 36 h. d) Quantification of IFN‐γ expressions by splenic CD4+ T cells after restimulating with 25 µg mL^−1^ HR121. e, f) Levels of IFN‐γ (Th1), TNF‐ɑ (Th1) and IL‐4 (Th2) in the supernatant of splenocytes after restimulating with 25 µg mL^−1^ HR121. g) Levels of IL‐17A (Th17) and IL‐22 (Th17) in the supernatant of splenocytes after restimulating with 25 µg mL^−1^ HR121. h, i) Representative flow cytometry dot plot and quantification of CD69 expression by splenic CD4+ (h) and CD8+ (i) T cells from immunized mice after restimulating with HR121. j, k) Anti‐HR121 titers of total IgG, IgG1 and IgG2a in sera from immunized mice. l) The titer ratios of IgG2a: IgG1 in indicated groups. Data were shown as mean±SD and statistical significance was analyzed with two‐tailed Student's *t*‐test and one‐way ANOVA with Tukey test, ns no significance, ^*^ *p*<0.05, ^**^ *p*<0.01, ^***^ *p*<0.001.

To further validate the prominent immunostimulatory properties observed, ipLNP with two doses (45 and 90 µg/mouse) was admixed with a model protein antigen HR121 and administered intramuscularly into mice thrice at two‐week intervals (**Figure** **9**a). HR121 is a highly conserved viral antigen reported in our previous study.^[^ ^33^ ^]^ To clearly show the adjuvant activity of ipLNP, the toll‐like receptor 3 (TLR3) agonist poly (I:C), a typical adjuvant, was adopted as a benchmark. TLR3 agonist was chosen, partially because its key signal trigger occurs on the lysosomal membrane, similar to that of ipLNP. Following the last immunization, immunized mice were sacrificed. Spleens and sera were harvested to investigate the antigen‐specific immune responses. No obvious change in spleen weight was observed between ipLNP and controls, indicating that a relatively low level of systemic inflammation was induced by ipLNP administration (Figure 9b). To analyze the HR121‐specific T cell immunity, the cytokine secretions in splenocytes from immunized mice were assessed after ex vivo stimulation with HR121 antigen.^[^ ^34^, ^35^ ^]^

Notably, ipLNP‐adjuvanted vaccination presented ≈4.5 fold higher number of IFN‐γ‐positive splenocytes compared with vaccines of poly (I:C)+HR121 and HR121 alone, as evidenced by ELISpot images (Figure 9c). Besides, this similar dose‐dependent trend of ipLNP was also observed in the evaluation of IFN‐γ+CD4+T cells percentage (Figure 9d) and IFN‐γ concentration (Figure 9e) in supernatants of splenocytes stimulated ex vivo with HR121. To further elucidate the Th1/Th2 profile of ipLNP‐adjuvanted responses, TNF‐α (Th1) and IL‐4 (Th2) concentrations were also determined (Figure 9f). And the higher secreted concentrations of Th1‐type cytokines (IFN‐γ, TNF‐α) over Th2‐type cytokines (IL‐4, near‐baseline) initially revealed a Th1‐skewed effector profile. In addition to Th1/Th2 responses, Th17 immunity, characterized by IL‐17A and IL‐22 secretion, is also a critical type for mucosal protection against pathogen infection (viruses and bacteria).^[^ ^36^ ^]^ Inspired by the significant enrichment of IL‐17 signaling in skin transcriptomic analysis, antigen‐triggered secretion level of IL‐17A and IL‐22 by splenocytes was assessed (Figure 9g). These results demonstrated an obvious antigen‐specific Th17 response induced by ipLNP‐adjuvanted vaccine, which was not observed with poly (I:C). Furthermore, the expression of T cell activation marker CD69 was determined to confirm the specific reactivity of T cells toward HR121 antigen. Consistent with cytokine results, ipLNP‐adjuvanted vaccination substantially improved the CD69 expression levels in splenic CD8+ and CD4+ T cells after ex vivo stimulation with HR121 antigen (Figure 9h,i), compared with poly (I:C) and HR121 alone. Especially, ipLNP with a dose of 90 µg elicited an ≈10% CD69+ CD8+ T cells in immunized spleen, yielding a 6‐fold increase relative to that of poly (I:C) (1.6%) and HR121 alone (1.4%). These results firmly proved the prominent induction activity of ipLNP toward cellular immunity.

With respect to humoral responses, sera collected from immunized mice were used to measure antigen‐specific antibody titers with ELISA, providing a further understanding of the adjuvant activity of ipLNP. Overall, ipLNP also elicited high‐level HR121‐specific humoral responses, showing an up to 160‐fold increase relative to HR121 immunization (Figure 9j–l). Moreover, a comparable reinforcement effect on IgG and IgG1 titer was observed between ipLNP and the benchmark adjuvant poly (I:C) (Figure 9j,k). Particularly, mice immunized with ipLNP‐adjuvanted vaccines substantially increased the level of IgG2a isotype (Th1‐associated) with an average titer of 59200, which was 9 folds over that of poly (I:C) (6400) and 148 folds in comparison with that of HR121 alone (400) (Figure 9k,l). This was consistent with the findings reagrding Th1‐skewed effector properties of ipLNP in T cell immunity analysis. In summary, the results above clarified that ipLNP possessed a potent adjuvant activity in inducing cellular (Th1 and Th17) and humoral responses.

### 2.7 ipLNP Displays Certain Tumor‐Suppressing Effects

**Figure 10:**
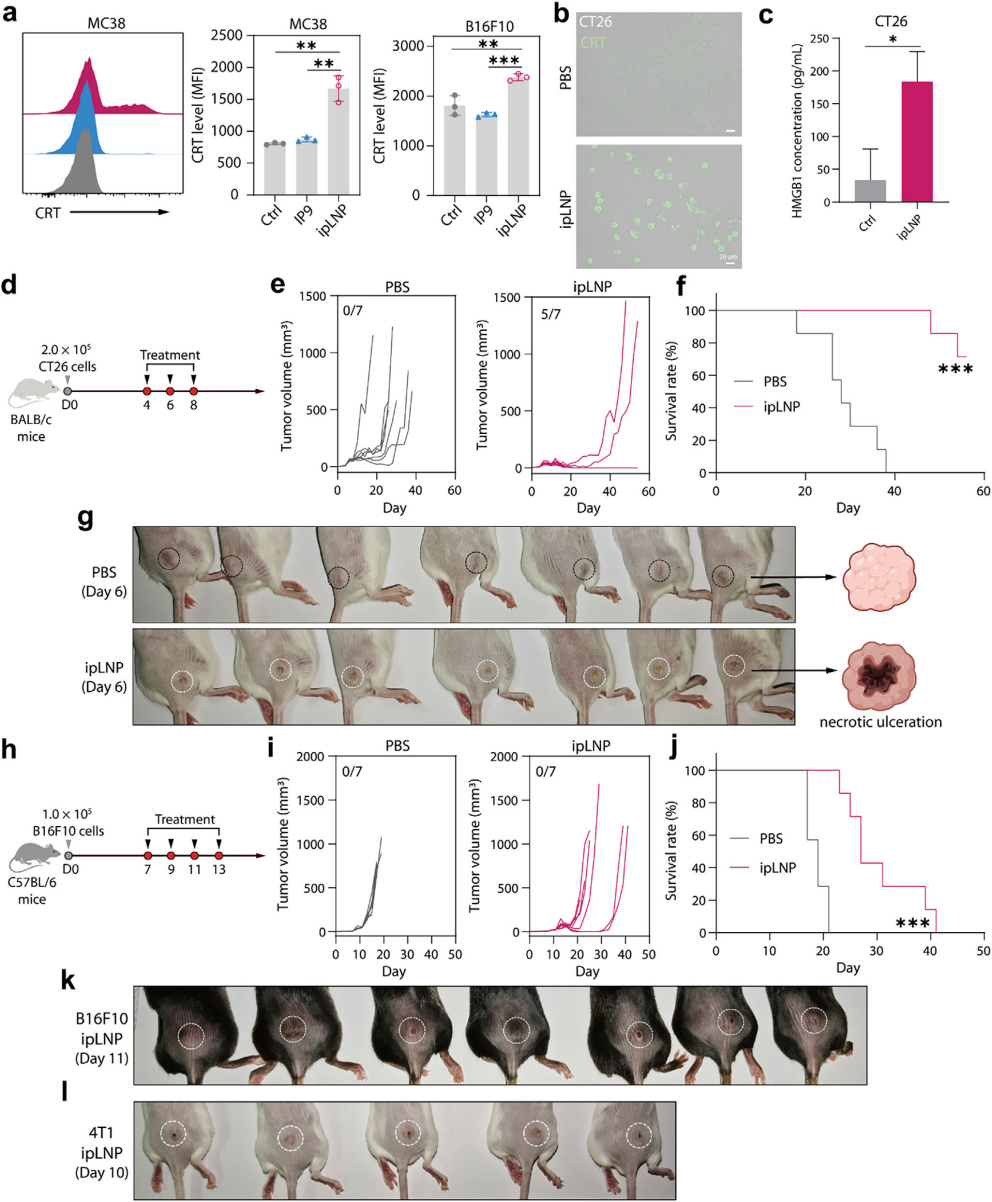
ipLNP displays certain tumor‐suppressing effects through inducing tumor necrosis. a) Flow cytometry analysis for the level of surface CRT on MC38 and B16F10. IP9 was used as a control. b) Microscope fluorescence images for the level of surface CRT on CT26. c) HMGB1 concentration in the supernatant of CT26 cells (n = 3). d) Administration scheme for mice with an established CT26 tumor. Mice were intratumorally injected with ipLNP (dose: D4 135 µg, D6 90 µg, D8 45 µg). e) Spider plots of tumor growth curves of individual mice administered with indicated treatments (5/7: complete responses mice versus total mice). f) Survival curves of CT26‐bearing mice administered with indicated treatments (n = 7 for PBS and ipLNP). g) Photographs of necrotic ulceration phenotype on CT26 tumor tissues at D6 after the first dose of ipLNP. h) Administration scheme for mice with an established B16F10 tumor. Mice were intratumorally injected with ipLNP at a dose of 90 µg four times. i) Spider plots of tumor growth curves of individual mice administered with indicated treatments. j) Survival curves of B16F10‐bearing mice administered with indicated treatments (n = 7 for PBS and ipLNP). k, l) Photographs of necrotic ulceration phenotype on B16F10 (k) and 4T1 (l) tumor tissues after treating with ipLNP. Data were shown as mean±SD and statistical significance was analyzed with two‐tailed Student's *t*‐test for CRT and HMGB1 analysis, and Mantel‐Cox test for survival percent, ^*^ *p*<0.05, ^**^ *p*<0.01, ^***^ *p*<0.001.

The broad and unique lytic cell death observed also motivated us to explore the therapeutic potential of ipLNP against cancer. Inducing immunogenic cell death (ICD) has been recognized as a robust strategy for cancer eradication. To clarify whether ipLNP elicits ICD, cell surface calreticulin (CRT), a crucial ICD biomarker, was measured using flow cytometry and fluorescence microscopy analysis. Surface‐exposed CRT can mediate dendritic cell phagocytosis (antigen presentation) and generate antitumor immunity.^[^ ^37^ ^]^ As indicated, ipLNP incubation significantly improved the level of surface‐exposed CRT in cells of MC38, B16F10, and CT26 (**Figure** **10**a,b), albeit to a limited extent, proving an involvement of ICD. Besides, IP9 alone did not have similar effects, again ‌highlighting the importance of nano formulation. To further confirm the observation, the release level of HMGB1 (another ICD biomarker) was also evaluated in the supernatant of CT26 cells using ELISA, and the results were consistent with those of CRT analysis (Figure 10c).

Based on this, an in vivo suppression evaluation of the established tumors was further performed. In the mouse colon carcinoma (CT26) model, intratumoral injection of ipLNP efficiently suppressed the growth of tumors, with over 70% mice displaying a complete response (5/7) (Figure 10d–f). Notably, after the first administration, obvious swelling and tumor cell necrosis occurred in the tumors of mice intratumorally treated with ipLNP, distinct from that of control mice (Figure 10g). This phenotype was consistent with skin abscess and lesion formation observed in the transcriptomic analysis, suggesting an involvement of ipLNP‐mediated ICD. Similar results were also observed in the mouse melanoma (B16F10) model. Tumor‐bearing mice treated with ipLNP had longer survival duration (median survival time: 27 days) than mice injected with PBS (median survival time: 19 days) (Figure 10h–j). Besides, swelling and tumor cell necrosis also appeared in the melanoma tumors of mice treated with ipLNP, displaying as a necrotic central collapse (Figure 10k). Interestingly, 4T1‐bearing mice treated with ipLNP displayed a highly similar phenotype (Figure 10l), validating the ubiquity of this observation. Briefly, the morphological alteration of tumor tissues was characterized by a distinctive central depression presenting as a necrotic ulceration. These results primarily demonstrated the application value of ipLNP in cancer therapy through inducing ICD. And the optimization for formulation and administration route of IP9‐based LNP is required to further improve the safety and efficacy.

## 3 Discussion and Conclusion

LNPs substantially enhance the bioavailability and targeted delivery of therapeutic agents, including mRNA, siRNA, and small molecules, by protecting payloads from degradation and improving cellular uptake‌. However, side effects and unknown involved mechanisms of LNPs raise concerns regarding their widespread application of LNPs. In this study, cargo‐free ipLNP is observed to induce a broad bubble morphology and lytic cell death in various cells, providing a unique clue for exploring the possible side effect mechanisms of LNP. ipLNP triggers a lysosome‐associated cell death through lysosomal membrane destabilization, and ipLNP‐induced cell death can be blocked by VE through maintaining lysosomal membrane integrity. Moreover, ipLNP displays the potential to function as a Th1/Th17‐directing vaccine adjuvant and therapeutic agent for cancer, indicating the feasibility of repurposing adverse effects.

In our previous study, STING agonist‐encapsulated ipLNP was found to induce necroptosis‐involved cell death.^[^ ^5^ ^]^ Clearly, cargo‐free ipLNP displays a different mechanism, which might be due to assembled structural differences derived from using citrate buffer without STING agonist. Despite the lysosome‐associated cell death described above, the precise mechanism from lysosomal membrane damage to plasma membrane swelling remains unclear. Our future work will focus on clarifying the mechanism using tools like CRISPRi. And there is a possible hypothesis that ipLNP leaked from the lysosomes into the cytosol further affects the integrity of the cell plasma membrane to induce cell death, which might account for the broad nature of ipLNP‐mediated cell death. Meanwhile, strategies modulating the lysosome membrane integrity with molecules like VE might provide side effect mitigation strategies for the currently used LNPs. Developing LNP with membrane fusion activity, avoiding the lysosome process, can be another direction for safer delivery vectors.

Revealing the side effects and involved mechanisms of LNP not only provides precise targets for adverse effects mitigation but also facilitates novel and potential applications of LNP by repurposing adverse effects. The adverse effects of repurposing refer to developing a new indication for existing drugs by balancing beneficial effects and adverse effects.^[^ ^38^ ^]^ Regarding ipLNP, the inflammation effect and ICD triggered by the damage of lysosome membrane exhibit prominent potentials to be developed as vaccine adjuvants and cancer therapeutics, beyond the previous carrier application. The remarkable immunostimulatory effect of ipLNP toward antigen‐specific Th1 and Th17 immunity highlights its potential as a novel adjuvant for virus and bacteria vaccine after proper optimization, due to the vital role of IFN‐γ and IL‐17 in mediating pathogen elimination. Moreover, screening helper lipid types and ratios,^[^ ^5^, ^39^ ^]^ exploring administration routes and loading anti‐inflammatory lipids (such as nitro‐oleic acid^[^ ^40^ ^]^) may be potential optimization strategies to attenuate inflammatory effect of ipLNP while preserving its adjuvant properties. The broad lytic cell death induced by ipLNP also provides an attractive choice for cancer therapy, especially for the treatment of drug‐resistant tumors. Despite showing therapeutic value, the therapeutic efficacy of ipLNP in vivo needs to be further improved to amplify the function of IP9.

In summary, our study reveals that the degree of lysosome membrane damage may be a checkpoint for cell homeostasis, inflammation, and cell death. Therefore, by screening ionizable lipids with different activities and mechanisms, disruption of the lysosome membrane can be precisely and gradationally regulated to achieve controllable and efficient delivery, immune activation, and cell death. This will effectively expand the application scope and fields of LNPs and not just their use as delivery vectors.

## 4 Experimental Section

### Materials

Heptadecan‐9‐yl 8‐((2‐hydroxyethyl)(6‐oxo‐6‐(undecyloxy)hexyl)amino)octanoate (SM‐102), 1,2‐Distearoyl‐sn‐glycero‐3‐phosphorylcholine (DSPC), (Z)‐(2R)‐3‐(((2‐Aminoethoxy)(hydroxy)phosphoryl)oxy)propane‐1,2‐diyl dioleate (DOPE), dimethyldioctadecylammonium (bromide salt) (DDAB), DMG‐PEG2000, necrostatin‐1 (Nec‐1), necrostatin 2 (Nec‐2), Liproxstatin‐1 (Lip‐1) and Chloroquine (CQ) were purchased from Bide Pharmatech. Ferrostatin‐1 (Fer‐1), vitamin E (VE), and Mito‐TEMPO were purchased from Sigma. Vitamin C (VC) was purchased from LABLEAD. Necrosulfonamide (NSA), Trolox, N‐Acetylcysteine (NAC), CA‐074, Pepstatin A (Pep‐A), E‐64, Deferiprone (DFe), 3‐methyladenine (3‐MA), BAPTA‐AM, caspase‐3 inhibitor Z‐DEVD‐FMK, and pan‐caspase inhibitor Z‐VAD‐FMK were purchased from MCE. HR121 protein was expressed by *E*. coli.^[^ ^33^ ^]^

### Cell Lines and Animals

CT26 (RRID: CVCL_7254), HeLa (RRID: CVCL_0030), MC38 (RRID:CVCL_B288), A549 (RRID: CVCL_0023), RAW‐ISG (RRID: CVCL_X595), Hep G2 (RRID: CVCL_0027) cells were cultured in DMEM medium (Gibco) containing 10% heat‐inactivated FBS (PAN), 100 U mL^−1^ penicillin (NCM Biotech), and 100 µg mL^−1^ streptomycin (NCM Biotech). B16F10 (RRID: CVCL_0159), 4T1 (RRID: CVCL_0125), B16‐OVA (RRID: CVCL_WM78), DC2.4 (RRID: CVCL_J409) cells were cultured in RPMI1640 medium (Gibco) with 10% FBS and the same supplements. The cell lines used were confirmed to be contamination free. Female BALB/c and C57BL/6 mice (purchased from Shanghai SLAC Laboratory Animal) were raised at the Laboratory Animal Center (SPF level) of Fujian Normal University with food and water ad libitum. Animal experiments were conducted according to the protocol approved by the Institutional Animal Care and Use Committee (IACUC) of Fujian Normal University (approval number: IACUC‐20230037).

### IP9 Synthesis and LNP Preparation

IP9 was synthesized according to our previous work.^[^ ^5^ ^]^ Cargo‐free LNP were also prepared using an ethanol dilution strategy. The LNP using DDAB as helper lipid was termed as ipLNP (IP9: DDAB: Chol: DMG‐PEG2000 = 60: 30: 40: 0.4, the same molar ratio as Siegwart's^[^ ^19^ ^]^ and our previous work^[^ ^5^ ^]^). For LNP containing DSPC and DOPE, IP9: DSPC/DOPE: Chol: DMG‐PEG2000 = 50/35: 10/16: 38.5/46.5: 1.5/2.5, respectively. The DSPC‐related molar ratio was set according to mRNA‐1273 (Moderna), and the DOPE‐related molar ratio was set according to Anderson's work,^[^ ^41^ ^]^ which was also a widely used ratio for DOPE‐containing LNP screening.^[^ ^42^ ^]^ The lipid mixture of IP9, helper lipid (DDAB, DSPC, DOPE), cholesterol, and DMG‐PEG2000 was resolved in ethanol, and the final concentration of IP9 was 7.4 mg mL^−1^ for DDAB mixture, 6.4 mg mL^−1^ for DSPC mixture, and 3.7 mg mL^−1^ for the DOPE mixture. The lipid solution above was mixed quickly with 10 mM citric acid/sodium citrate buffer (pH = 4.4) at a volume ratio of 1:3. After 15 min, the mixture solution was diluted with three‐fold volume of PBS for the following in vitro and in vivo experiments.

### Phenotypic Analysis of Cell Death

Cells were seeded on 8‐well confocal imaging chamber overnight and incubated with ipLNP at the indicated concentrations. When obvious cell bubbles were observed, cells were stained with Annexin V‐mCherry/SYTOX Green (Beyotime) according to the manufacturer's instructions and subjected to CLSM imaging (ZEISS LSM 780) and flow cytometry analysis (Agilent NovoCyte 3130). To analyze the dynamic changes in cell morphology, B16F10, 4T1, and DC2.4 cells were seeded on a 96‐well plate overnight and simultaneously added with ipLNP (5 µg mL^−1^) and SYTOX Green (1:200 dilution). The plate was subjected to a high‐throughput microplate imager for high‐content analysis (Operetta CLS, Perkin Elmer), maintained at 37 °C and 5% CO_2_. The images of bright field and FITC channel fluorescence were continually recorded every 10 min. To evaluate cell rupture, the cell culture medium after ipLNP incubation was used for LDH level determination with the CytoTox 96 cytotoxicity assay (Promega) according to the manufacturer's instructions. Cell viability was assessed using Cell Counting Kit‐8 (CCK‐8, Beyotime).

### Osmoprotectant PEG4000/8000 Evaluation

CT26 cells were seeded on a 96‐well plate and first incubated with ipLNP (5 µg mL^−1^) for 4 h. After that, the wells were directly added with PEG4000 (Sigma, 0.1 g mL^−1^) and PEG8000 (Sigma, 0.1 g mL^−1^). LDH and cell viability were tested after incubation for 9 h and 12 h, respectively.

### Intracellular ROS Level and Lipid Peroxidation

For total ROS level and lipid peroxidation analysis, cells were treated with indicated groups for 4.5–6.5 h and then stained with DCFH‐DA (Beyotime, 0.5 µm) for 0.5 h, and BODIPY 581/591 C11 (Invitrogen, 2.5 µm). After washing, cells were analyzed with BD FACSymphony A5 flow cytometry. Data was processed with FlowJo (v10.8.1). For ROS type analysis, CT26 cells were seeded on 8‐well confocal imaging chamber and treated with SNP (SM‐102 12.96 µg mL^−1^) and ipLNP (IP9 9 µg mL^−1^). Cells were then stained with SOSG (Beyotime, 5 µm) for 1 h, DHE (Beyotime, 5 µm) for 0.5 h. After washing, cells were imaged with ZEISS LSM 780.

### Caspase‐3 Activation and GSDME Cleavage

Caspase‐3 activation was assessed by GreenNuc Caspase‐3 Assay Kit (Beyotime). CT26 cells were seeded on 8‐well confocal imaging chamber and incubated with SNP (SM‐102 16.2 µg mL^−1^), PAC‐1 (caspase‐3 activator, Beyotime, 100 µm), and ipLNP (IP9 11.25 µg mL^−1^) for 4 h. After that, GreenNuc caspase‐3 substrate (DEVD‐DNA dye conjugate) was added for CLSM imaging (ZEISS LSM 780) with FITC channel. To further quantify the fluorescence level, CT26 cells were seeded on a black 96‐well plate, treated with a similar process described above, and the fluorescence intensity was tested with a fluorescence microplate reader. For GSDME cleavage analysis, HeLa and CT26 cells were seeded on 6‐well plates at a density of 70%–80%. The cells were lysed with SDS‐loading buffer and collected for detecting GSDME cleavage with western blot analysis. Anti‐GSDME N‐terminal antibody (abcam, Rabbit, 1:5000), and HRP‐conjugated Goat Anti‐Rabbit IgG (Proteintech, 1:8000) were used for GSDME full‐length and N‐terminal detection, and the western blot images were captured with Cytiva Amersham ImageQuant 800.

### Transcriptomic Analysis of Cell and Skin

For cell RNA‐Seq analysis, HeLa cells were seeded on 6‐well plate (7 × 10^5^ cells) overnight and incubated with ipLNP (IP9 11.25 µg mL^−1^). When a small number of cells exhibited the bubble phenotype, wells were washed and then filled with 1 mL Trizol (Takara) to lyse cells. The samples were quickly frozen with liquid nitrogen and analyzed by BGI Genomics. For skin RNA‐Seq analysis, female BALB/c mice were intradermally injected with ipLNP (90 µg per mouse) and PBS (control). 24 h post‐injection, mice were sacrificed and skins (diameter 1 cm) at the injection site were collected and quickly frozen with liquid nitrogen. The skin samples were analyzed by Novogene.

### Cell Death Inhibitors Screening

To clarify the mechanism involved in ipLNP‐induced cell death, CT26, MC38, 4T1, and B16F10 cells were pretreated with inhibitors targeting various cell death pathways for 12 h, and then ipLNP and fresh inhibitors were added into wells to replace the previous medium for another 24 h. The inhibitors used include Nec‐1 (10 µM), Nec‐2 (10 µM), NSA (2.5 µM), DFe (1 µM), Fer‐1 (20 µM), Lip‐1 (1 µM), CQ (10 µm), 3‐MA (1 mM), caspase‐3 inhibitor Z‐DEVD‐FMK (50 µm), pan‐caspase inhibitor Z‐VAD‐FMK (50 µm), and VE (75 µm). As for lysosomal contents and ROS related inhibitors, CT26 cells were pretreated with inhibitors including CA‐074 (20 µm), Pep‐A (20 µm), E‐64 (10 µg mL^−1^), BAPTA‐AM (1 µm), Mito‐TEMPO (500 µm), NAC (7.5 mM), VC (100 µm), Trolox (100 µm), and VE (75 µm) for 12 h. And ipLNP and fresh inhibitors were added into wells to replace the previous medium and incubated for indicated hours. CCK‐8 cell viability assay, cell morphology imaging, LDH release, and intracellular ROS level were adopted to analyze the rescue efficacy.

### Cellular Colocalization and Dynamic Distribution

To visualize the cellular distribution, ipLNP was labeled with fluorescent DiI using the lipid mixture containing 4 mol% DiI for the following ethanol dilution preparation. CT26, DC2.4 cells were seeded on an 8‐well confocal imaging chamber overnight. DiI‐labeled ipLNP and VE‐related groups were added and incubated for the indicated hours. For colocalization analysis, cells incubated with DiI‐labeled ipLNP (6.75 µg mL^−1^) were further stained with Lyso‐Tracker Green probe (lysosome, Beyotime, 1 µm), Mito‐Tracker Green (mitochondrion, Beyotime, 1.67 µm), ER‐Tracker Green (ER, Beyotime, 400 µm), and Hoechest according to the manufacturer's protocol. The samples were subjected to CLSM imaging (ZEISS LSM 780) using FITC, DiI, and DAPI channels, and the fluorescence intensity was quantified by ImageJ.

### Lysosome Acidification

CT26 cells were seeded on the 96‐well plate and 8‐well confocal imaging chamber overnight. After incubation with ipLNP and controls, cells in 96‐well plate were stained with Lyso‐Tracker Green probe (375 nM, 1 h) for flow cytometry analysis (Agilent NovoCyte 3130), and cells in a chamber were stained with Lyso‐Tracker Red probe (375 nM, 1 h) for CLSM imaging (ZEISS LSM 780).

### Macrophage Activation

Mouse macrophage cell line RAW‐ISG was seeded on a 24‐well plate overnight, and ipLNP was added with indicated concentrations. After incubation for 24 h, cells were collected and stained with ɑCD40‐APC (clone 3/23, Biolegend) and ɑCD86‐FITC (clone GL‐1, Biolegend) for 0.5–1 h on ice and analyzed in BD FACSymphony A5 flow cytometry.

### Animal Immunization

To evaluate the adjuvant effect, ipLNP was first prepared according to the process described above and further added to SARS‐CoV‐2 antigen HR121 in PBS buffer. The mixture of ipLNP and HR121 was intramuscularly administered into the hind legs of BALB/c mice (6‐week‐old) with a dose of 45/90 µg ipLNP and 10 µg HR121. The typical adjuvant poly (I:C) (InvivoGen, 50 µg per mouse) was mixed with HR121 (10 µg per mouse) and intramuscularly administered into the hind legs of BALB/c mice. The immunization was performed three times biweekly, and samples of spleen, sera were collected one week after the last immunization.

### Antigen‐Specific T Cell

Spleens harvested from immunized mice were mechanically dissociated and filtered through 40 µm cell strainers to generate single‐cell suspensions. Splenocytes were lysed using NH_4_Cl buffer, followed by splenocyte quantification. For ELISpot analysis, 5 × 10^5^ splenocytes were seeded on an IFN‐γ‐precoated 96‐well plate (Dakewe Biotech). HR121 proteins were added to wells (final concentration: 50 µg mL^−1^) and cells were stimulated for 36 h. PMA+ionomycin was used as a positive control. IFN‐γ spots were developed according to the ELISpot Kit instructions and read in the Mabtech EliSpot Reader. For intracellular cytokine staining of CD4+ T cells, splenocytes were seeded into 24‐well plates and stimulated with 25 µg mL^−1^ HR121 for 3 h. Protein transport inhibitors Brefeldin A and Monensin (eBioscience, 1:1000 dilution) were then added, and cells were incubated for an additional 8 h. After washing, surface markers PerCP/Cyanine5.5‐anti‐CD3 (17A2, Biolegend) and BV421‐anti‐CD4 (RM4‐4, Biolegend) were labeled. Cells were subsequently fixed, permeabilized (eBioscience), and intracellularly stained with PE‐anti‐IFN‐γ (XMG1.2, Biolegend) and isotype controls for flow cytometry analysis (BD FACSymphony A5). For T cell activation analysis, splenocytes were stimulated with 25 µg mL^−1^ HR121 for 72 h. Post incubation, cells and supernatants were separated by centrifugation. Cells were stained with PerCP/Cyanine5.5‐anti‐CD3, BV421‐anti‐CD4, APC‐anti‐CD8α (53‐6.7, Biolegend), PE‐anti‐CD69 (H1.2F3, Biolegend), and isotype controls (BioLegend). Data was processed with FlowJo (v10.8.1). IFN‐γ, TNF‐α, IL‐4, IL‐17A, and IL‐22 levels in supernatants of antigen‐stimulated splenocytes were quantified using ELISA kits (Dakewe Biotech) according to the manufacturer's protocol.

### Antigen‐Specific Antibody

Blood samples from immunized mice were centrifuged to isolate sera. HR121 protein (2 µg mL^−1^) was dissolved in coating buffer (0.1 M NaHCO_3_, pH 9.6). A total of 100 µL well^−1^ of antigen solution was added to 96‐well plates (Corning Costar) and incubated overnight at 4 °C. Then, plates were blocked with 0.25% gelatin in PBS for 1 h at room temperature. After washing, diluted serum samples were then added (100 µL well^−1^) and incubated for 1 h. After washing, rabbit anti‐mouse IgG‐HRP, IgG1‐HRP, and IgG2a‐HRP antibodies (Abcam, 1:2000 dilution in PBS, 100 µL well^−1^) were added. Following 1 h incubation, plates were washed and developed with 3,3′,5,5′‐tetramethylbenzidine (TMB, Beyotime, 200 µL well^−1^). Reactions were terminated with 50 µL well^−1^ of 2 M H_2_SO_4_. Absorbance at 450 nm was measured using a FLUOstar Optima microplate reader. Antibody titers were defined as the highest serum dilution yielding an absorbance value ≥0.1 above the blank (unimmunized serum control).

### Immunogenic Cell Death

For flow cytometry analysis, cells treated with ipLNP were collected and stained with anti‐CRT polyclonal antibody (Proteintech, Rabbit, 1:200) and CoraLite488‐conjugated Goat anti‐Rabbit IgG(H+L) (Proteintech, 1:200) when an obvious phenotype occurred. For fluorescence microscope analysis, cells were seeded on coverslips in a 6‐well plate (70–80% confluence) and incubated with ipLNP (10 µg mL^−1^). The coverslips were subjected to immunofluorescence staining with antibodies mentioned (anti‐CRT polyclonal antibody 1:200, CoraLite488‐conjugated Goat anti‐Rabbit IgG(H+L) 1:1000) in flow cytometry analysis until obvious phenotypes occurred, and images were captured with Keyence BZ‐X810 fluorescence microscope.

### In Vivo Anti‐Tumor Study

Female C57BL/6 mice (5‐week‐old) were inoculated subcutaneously into the right flank with 1.5 × 10⁵ B16F10 cells suspended in 100 µL of serum‐free RPMI1640 medium. Female BALB/c mice (5‐week‐old) were inoculated subcutaneously into the right flank with 1.0 × 10⁵ CT26 cells suspended in 100 µL of serum‐free DMEM medium. Tumor growth was monitored every other day using digital calipers, with volume calculated as L×W×W×0.5, where L and W represent the longest and perpendicular tumor diameters, respectively. Mice were humanely euthanized when tumors reached a maximum diameter of 15 mm. Mice with established tumors were injected with ipLNP at indicated doses through an intratumoral route.

### Statistical Analysis

Data were presented as mean ± standard deviation (SD). All experiments included at least three independent replicates. Statistical analyses were performed using GraphPad Prism software (version 8.0.2, GraphPad Inc.), applying one‐way ANOVA with Tukey's post‐hoc test for multi‐group comparisons or Student's *t*‐test for pairwise comparisons. Statistical significance was set as follows: ns, no significance, ^*^ *p*<0.05, ^**^ *p*<0.01, ^***^ *p*<0.001.

## Conflict of Interest

The authors declare no conflict of interest.

## Supporting information



Supporting Information

Supplemental Video 1

Supplemental Video 2

Supplemental Video 3

## Data Availability

The data that support the findings of this study are available from the corresponding author upon reasonable request.

## References

[1] X. Hou , T. Zaks , R. Langer , Y. Dong , Nat. Rev. Mater. 2021, 6, 1078. 34394960 10.1038/s41578-021-00358-0 PMC8353930

[2] P. R. Cullis , P. L. Felgner , Nat. Rev. Drug Discov. 2024, 23, 709. 38965378 10.1038/s41573-024-00977-6

[3] B. Hu , L. Zhong , Y. Weng , L. Peng , Y. Huang , Y. Zhao , X.‐J. Liang , Signal Transduct. Target. Ther. 2020, 5, 101. 32561705 10.1038/s41392-020-0207-x PMC7305320

[4] V. Madigan , F. Zhang , J. E. Dahlman , Nat. Rev. Drug Discov. 2023, 22, 875. 37723222 10.1038/s41573-023-00762-x

[5] J.‐J. Wu , G.‐J. Chen , C.‐Y. Fan , F. Shen , Y. Yang , W. Pang , Z.‐N. Zhao , H.‐X. Guan , H. Wu , Y. Lu , Y.‐J. Fu , Q. Chen , Y.‐T. Zheng , S. Ouyang , Adv. Funct. Mater. 2023, 33, 2306010.

[6] Y. Li , Z. Ye , H. Yang , Q. Xu , Acta Pharm. Sin. B 2022, 12, 2624. 35755280 10.1016/j.apsb.2022.04.013 PMC9214058

[7] Q. Cheng , T. Wei , L. Farbiak , L. T. Johnson , S. A. Dilliard , D. J. Siegwart , Nat. Nanotechnol. 2020, 15, 313. 32251383 10.1038/s41565-020-0669-6 PMC7735425

[8] M. Hadjidemetriou , M. Mahmoudi , K. Kostarelos , Nat. Rev. Mater. 2024, 9, 219.

[9] D. Bitounis , E. Jacquinet , M. A. Rogers , M. M. Amiji , Nat. Rev. Drug Discov. 2024, 23, 281. 38263456 10.1038/s41573-023-00859-3

[10] K. J. Hassett , K. E. Benenato , E. Jacquinet , A. Lee , A. Woods , O. Yuzhakov , S. Himansu , J. Deterling , B. M. Geilich , T. Ketova , C. Mihai , A. Lynn , I. McFadyen , M. J. Moore , J. J. Senn , M. G. Stanton , Ö. Almarsson , G. Ciaramella , L. A. Brito , Mol. Ther. Nucleic Acids 2019, 15, 1. 30785039 10.1016/j.omtn.2019.01.013 PMC6383180

[11] H. Y. Xue , S. Liu , H. L. Wong , Nanomedicine (Lond) 2014, 9, 295. 24552562 10.2217/nnm.13.204 PMC4095781

[12] M. Sedic , J. J. Senn , A. Lynn , M. Laska , M. Smith , S. J. Platz , J. Bolen , S. Hoge , A. Bulychev , E. Jacquinet , V. Bartlett , P. F. Smith , Vet. Pathol. 2018, 55, 341. 29191134 10.1177/0300985817738095

[13] D. W. Brown , P. Wee , P. Bhandari , A. Bukhari , L. Grin , H. Vega , M. Hejazi , D. Sosnowski , J. Ablack , E. K. Clancy , D. Pink , J. Kumar , M. P. Solis Ares , S. Lamb , R. Quevedo , B. Rawal , F. Elian , N. Rana , L. Morales , N. Govindasamy , B. Todd , A. Delmage , S. Gupta , N. McMullen , D. MacKenzie , P. H. Beatty , H. Garcia , M. Parmar , J. Gyoba , C. McAllister , M. Scholz , R. Duncan , A. Raturi , J. D. Lewis , Cell 2024, 187, 5357. 39260374 10.1016/j.cell.2024.07.023

[14] M.‐G. Alameh , I. Tombácz , E. Bettini , K. Lederer , S. Ndeupen , C. Sittplangkoon , J. R. Wilmore , B. T. Gaudette , O. Y. Soliman , M. Pine , P. Hicks , T. B. Manzoni , J. J. Knox , J. L. Johnson , D. Laczkó , H. Muramatsu , B. Davis , W. Meng , A. M. Rosenfeld , S. Strohmeier , P. J. C. Lin , B. L. Mui , Y. K. Tam , K. Karikó , A. Jacquet , F. Krammer , P. Bates , M. P. Cancro , D. Weissman , E. T. Luning Prak , D. Allman , M. Locci , N. Pardi , Immunity 2021, 54, 2877. 34852217 10.1016/j.immuni.2021.11.001 PMC8566475

[15] S. Ndeupen , Z. Qin , S. Jacobsen , A. Bouteau , H. Estanbouli , B. Z. Igyarto , iScience 2021, 24, 103479. 34841223 10.1016/j.isci.2021.103479 PMC8604799

[16] Y. Zhu , J. Ma , R. Shen , J. Lin , S. Li , X. Lu , J. L. Stelzel , J. Kong , L. Cheng , I. Vuong , Z.‐C. Yao , C. Wei , N. M. Korinetz , W. H. Toh , J. Choy , R. A. Reynolds , M. J. Shears , W. J. Cho , N. K. Livingston , G. P. Howard , Y. Hu , S. Y. Tzeng , D. J. Zack , J. J. Green , L. Zheng , J. C. Doloff , J. P. Schneck , S. K. Reddy , S. C. Murphy , H.‐Q. Mao , Nat. Biomed. Eng. 2024, 8, 544. 38082180 10.1038/s41551-023-01131-0 PMC11162325

[17] Y. Lee , M. Jeong , J. Park , H. Jung , H. Lee , Exp. Mol. Med. 2023, 55, 2085. 37779140 10.1038/s12276-023-01086-x PMC10618257

[18] J. J. Wu , L. Zhao , H. G. Hu , W. H. Li , Y. M. Li , Med. Res. Rev. 2020, 40, 1117. 31793026 10.1002/med.21649

[19] S. Liu , Q. Cheng , T. Wei , X. Yu , L. T. Johnson , L. Farbiak , D. J. Siegwart , Nat. Mater. 2021, 20, 701. 33542471 10.1038/s41563-020-00886-0 PMC8188687

[20] J. Wu , H. Wang , P. Gao , S. Ouyang , Acta Pharm. Sin. B 2024, 14, 4195. 39525577 10.1016/j.apsb.2024.06.026 PMC11544194

[21] L. Galluzzi , O. Kepp , F. K. Chan , G. Kroemer , Annu. Rev. Pathol. 2017, 12, 103. 27959630 10.1146/annurev-pathol-052016-100247 PMC5786374

[22] N. Kayagaki , O. S. Kornfeld , B. L. Lee , I. B. Stowe , K. O'Rourke , Q. Li , W. Sandoval , D. Yan , J. Kang , M. Xu , J. Zhang , W. P. Lee , B. S. McKenzie , G. Ulas , J. Payandeh , M. Roose‐Girma , Z. Modrusan , R. Reja , M. Sagolla , J. D. Webster , V. Cho , T. D Andrews , L. X. Morris , L. A. Miosge , C. C. Goodnow , E. M. Bertram , V. M. Dixit , Nature 2021, 591, 131. 33472215 10.1038/s41586-021-03218-7

[23] X. Chen , W.‐T. He , L. Hu , J. Li , Y. Fang , X. Wang , X. Xu , Z. Wang , K. Huang , J. Han , Cell Res. 2016, 26, 1007. 27573174 10.1038/cr.2016.100 PMC5034106

[24] L. Pedrera , R. A. Espiritu , U. Ros , J. Weber , A. Schmitt , J. Stroh , S. Hailfinger , S. von Karstedt , A. J. García‐Sáez , Cell Death Differ. 2021, 28, 1644. 33335287 10.1038/s41418-020-00691-x PMC8167089

[25] M. Liu , L. Huang , W. Zhang , X. Wang , Y. Geng , Y. Zhang , L. Wang , W. Zhang , Y.‐J. Zhang , S. Xiao , Y. Bao , M. Xiong , J. Wang , Nat. Nanotechnol. 2022, 17, 541. 35332294 10.1038/s41565-022-01085-5

[26] X. Hu , J. Li , Y. Zhang , M. Xiong , H. Zhang , Y. Yuan , Biomater. Sci. 2023, 11, 1451. 36602031 10.1039/d2bm01892f

[27] J. Li , F. Cao , H.‐L. Yin , Z.‐J. Huang , Z.‐T. Lin , N. Mao , B. Sun , G. Wang , Cell Death Dis. 2020, 11, 88. 32015325 10.1038/s41419-020-2298-2 PMC6997353

[28] G. Du , L. B. Healy , L. David , C. Walker , T. J. El‐Baba , C. A. Lutomski , B. Goh , B. Gu , X. Pi , P. Devant , P. Fontana , Y. Dong , X. Ma , R. Miao , A. Balasubramanian , R. Puthenveetil , A. Banerjee , H. R. Luo , J. C. Kagan , S. F. Oh , C. V. Robinson , J. Lieberman , H. Wu , Nature 2024, 630, 437. 38599239 10.1038/s41586-024-07373-5 PMC11283288

[29] B. Chen , Y. Yan , Y. Yang , G. Cao , X. Wang , Y. Wang , F. Wan , Q. Yin , Z. Wang , Y. Li , L. Wang , B. Xu , F. You , Q. Zhang , Y. Wang , Nat. Nanotechnol. 2022, 17, 788. 35606443 10.1038/s41565-022-01125-0

[30] Y. Chen , S. Zhu , T. Liao , C. Wang , J. Han , Z. Yang , X. Lu , Z. Hu , J. Hu , X. Wang , M. Gu , R. Gao , K. Liu , X. Liu , C. Ding , S. Hu , X. Liu , PLoS Pathog. 2024, 20, 1011981. 10.1371/journal.ppat.1011981 PMC10866534 38354122

[31] M. Cao , X. Luo , K. Wu , X. He , Signal Transduct. Target. Ther. 2021, 6, 379. 34744168 10.1038/s41392-021-00778-y PMC8572923

[32] Z. Yang , Z. Min , B. Yu , Int. Rev. Immunol. 2020, 39, 292. 32423322 10.1080/08830185.2020.1768251

[33] W. Pang , Y. Lu , Y.‐B. Zhao , F. Shen , C.‐F. Fan , Q. Wang , W.‐Q. He , X.‐Y. He , Z.‐K. Li , T.‐T. Chen , C.‐X. Yang , Y.‐Z. Li , S.‐X. Xiao , Z.‐J. Zhao , X.‐S. Huang , R.‐H. Luo , L.‐M. Yang , M. Zhang , X.‐Q. Dong , M.‐H. Li , X.‐L. Feng , Q.‐C. Zhou , W. Qu , S. Jiang , S. Ouyang , Y.‐T. Zheng , Cell Res. 2022, 32, 1068. 36357786 10.1038/s41422-022-00746-3 PMC9648449

[34] J.‐J. Wu , F.‐Y. Chen , B.‐B. Han , H.‐Q. Zhang , L. Zhao , Z.‐R. Zhang , J.‐J. Li , B.‐D. Zhang , Y.‐N. Zhang , Y.‐X. Yue , H.‐G. Hu , W.‐H. Li , B. Zhang , Y.‐X. Chen , D.‐S. Guo , Y.‐M. Li , CCS Chem. 2023, 5, 885.

[35] J.‐J. Wu , L. Zhao , B.‐B. Han , H.‐G. Hu , B.‐D. Zhang , W.‐H. Li , Y.‐X. Chen , Y.‐M. Li , Chem. Commun. 2021, 57, 504. 10.1039/d0cc06959k 33331360

[36] I. A. Hajam , C.‐M. Tsai , C. Gonzalez , J. R. Caldera , M. Lázaro Díez , X. Du , A. Aralar , B. Lin , W. Duong , G. Y. Liu , Nat. Commun. 2024, 15, 10335. 39681568 10.1038/s41467-024-54644-w PMC11649901

[37] P. Liu , L. Zhao , O. Kepp , G. Kroemer , Methods Enzymol. 2020, 632, 1. 32000891 10.1016/bs.mie.2019.05.011

[38] Y. Xia , M. Sun , H. Huang , W. L. Jin , Signal Transduct. Target. Ther. 2024, 9, 92. 38637540 10.1038/s41392-024-01808-1 PMC11026526

[39] M. Vadovics , W. Zhao , E. F. Daley , K. Lam , O. Daly , K. Rashid , H. R. Lee , P. Schreiner , K. A. Lundgreen , B. T. Gaudette , V. V. Shuvaev , E. Arguiri , H. Muramatsu , A. Sárközy , T. Mdluli , J. Xu , X. Han , N. De Luna , D. Castaño , E. Bettini , E. Ábrahám , Z. Lipinszki , G. Carlucci , A. H. Bansode , K. Nguyen , T. M. Le , T. Luu , V. R. Muzykantov , P. Bates , D. Allman , M. J. Mitchell , M. Locci , C. G. Radu , J. Heyes , N. Pardi , Nat. Nanotechnol. 2025. 10.1038/s41565-025-01958-5 PMC13150451 40550975

[40] M. N. Patel , S. Tiwari , Y. Wang , S. O'Neill , J. Wu , S. Omo‐Lamai , C. Espy , L. S. Chase , A. Majumder , E. Hoffman , A. Shah , A. Sárközy , J. Katzen , N. Pardi , J. S. Brenner , Nat. Biotechnol. 2025. 10.1038/s41587-025-02556-5 PMC12235526 39910195

[41] B. Li , A. Y. Jiang , I. Raji , C. Atyeo , T. M. Raimondo , A. G. R. Gordon , L. H. Rhym , T. Samad , C. MacIsaac , J. Witten , H. Mughal , T. M. Chicz , Y. Xu , R. P. McNamara , S. Bhatia , G. Alter , R. Langer , D. G. Anderson , Nat. Biomed. Eng. 2025, 9, 167. 37679571 10.1038/s41551-023-01082-6

[42] J. R. Melamed , S. S. Yerneni , M. L. Arral , S. T. LoPresti , N. Chaudhary , A. Sehrawat , H. Muramatsu , M.‐G. Alameh , N. Pardi , D. Weissman , G. K. Gittes , K. A. Whitehead , Sci. Adv. 2023, 9, ade1444. 10.1126/sciadv.ade1444 PMC9882987 36706177

