# Supplementary material for: Novel Swelling‐Lytic Cell Death Triggered by Cargo‐Free Ionizable Lipid Nanoparticles
Source: Adv Sci (Weinh). 2025 Aug 7;12(40):e09208. doi: 10.1002/advs.202509208 (PMC12561346; doi:10.1002/advs.202509208)
Supplement: Supplementary file 1 — Supporting Information [file ADVS-12-e09208-s004.docx]

**Supplementary Information for**

**Novel Swelling-Lytic Cell Death Triggered by Cargo-Free Ionizable Lipid Nanoparticles**

*Junjun Wu, Zhennan Zhao, Hongsheng Wu,* *Sihuang Lin, Lin Huang, Guanjie Chen, Yi Yang, Hong Wang, Huijie Yan, Yonghui Shi, Liuyu Zhu, Guosheng Hu, Liling Zheng*,* *Songying Ouyang**

J. Wu, Z. Zhao, H. Wu, L. Huang, G. Chen, Y. Yang, H. Wang, H. Yan, Y. Shi, L. Zhu, G. Hu, S. Ouyang

Key Laboratory of Microbial Pathogenesis and Interventions of Fujian Province University Provincial University, the Key Laboratory of Innate Immune Biology of Fujian Province, Biomedical Research Center of South China, College of Life Sciences, Fujian Normal University, Fuzhou 350117, China.

Email: [ouyangsy@fjnu.edu.cn](mailto:ouyangsy@fjnu.edu.cn)

S. Lin, L. Zheng

First Hospital of Quanzhou Affiliated with Fujian Medical University, Quanzhou 362000, China.

Email: [zll@fjmu.edu.cn](mailto:zll@fjmu.edu.cn)

J. Wu, Z. Zhao, H. Wu and S. Lin contributed equally to this work.

**This PDF file includes:**

Supplementary Figure 1-9


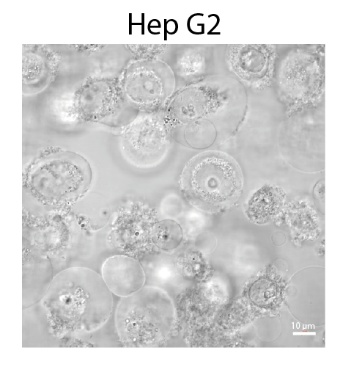


**Figure S1.** Representative image of swelling bubbles in Hep G2 cell line treated with ipLNP.


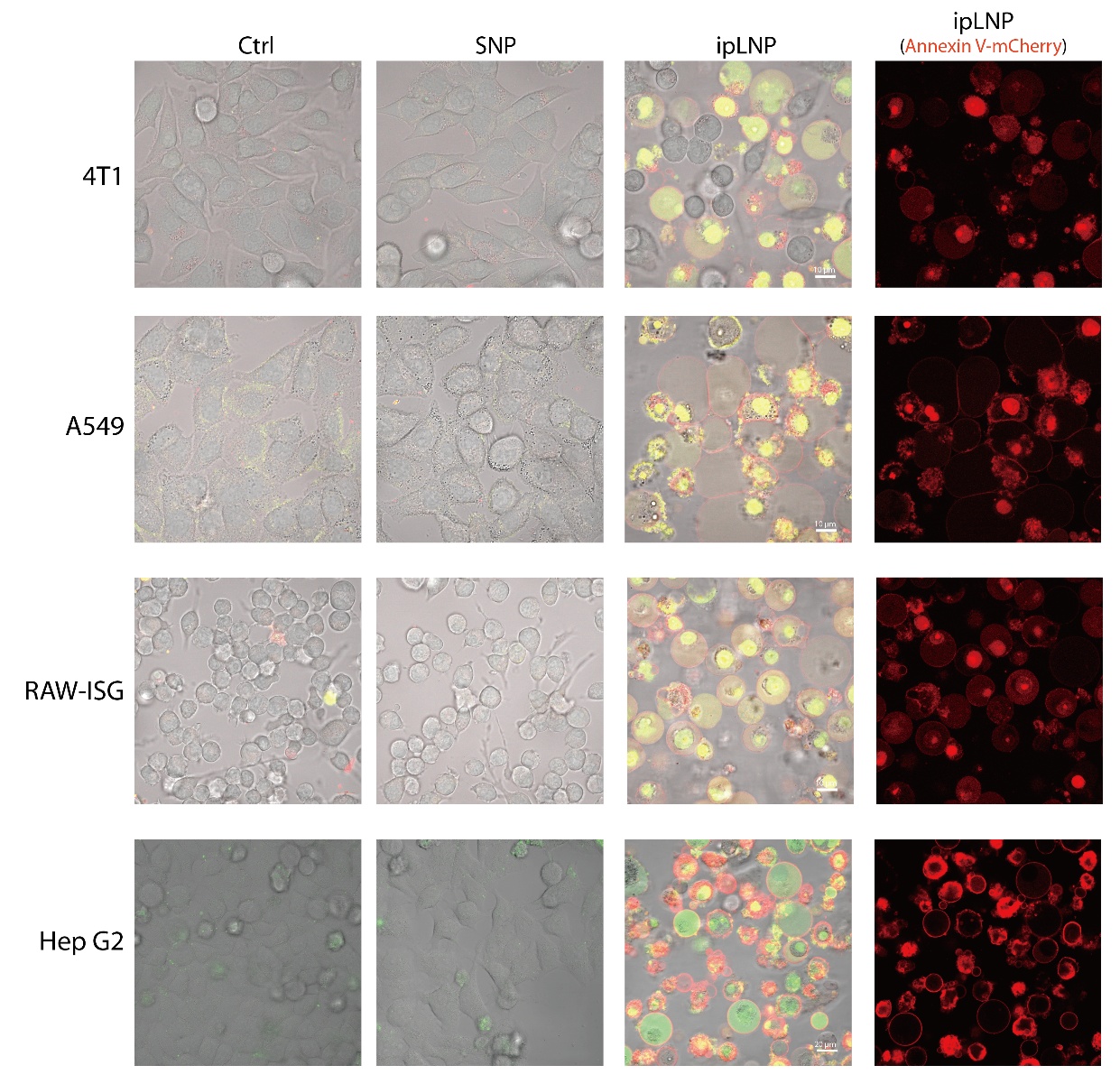


**Figure S2.** Confocal images of cells stained with Annexin V-mCherry/SYTOX Green after incubation with ipLNP and SNP.


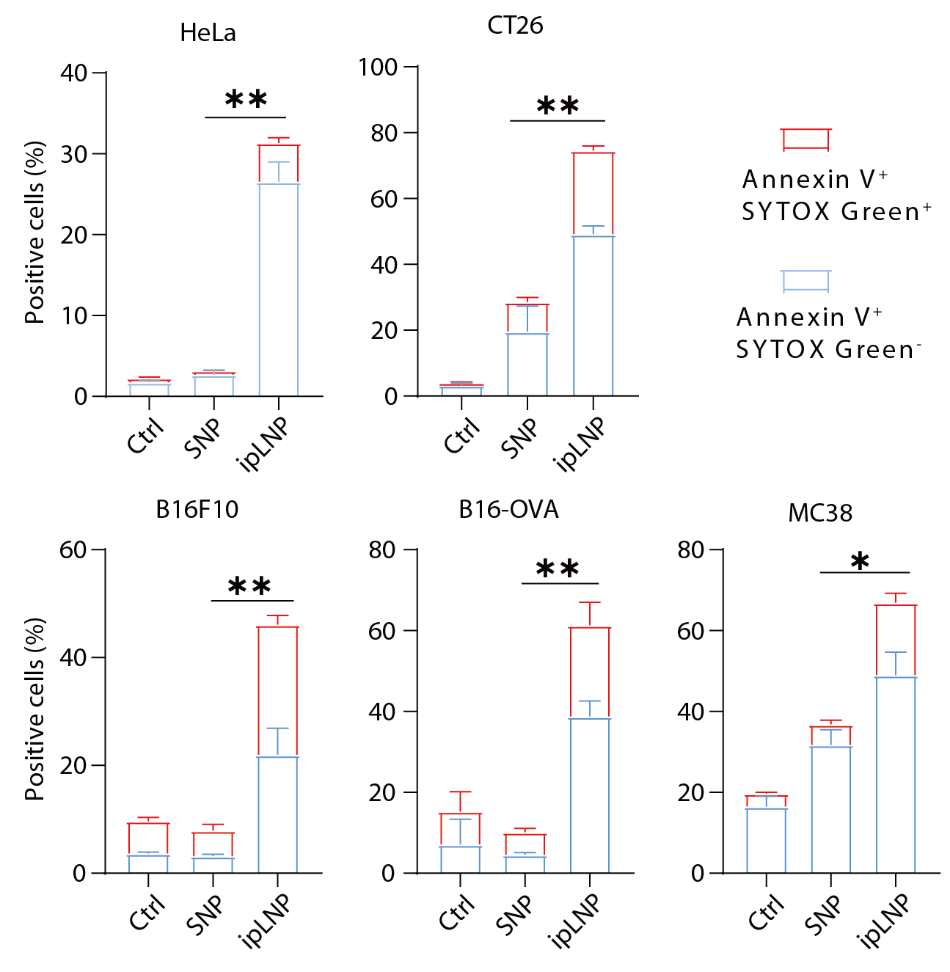


**Figure S3.** Quantification of double-positive and single-positive staining cells in ipLNP-treated cells. Data were shown as mean±SD and statistical significance was analyzed with two-tailed Student’s t-test, **p*<0.05, ***p*<0.01.


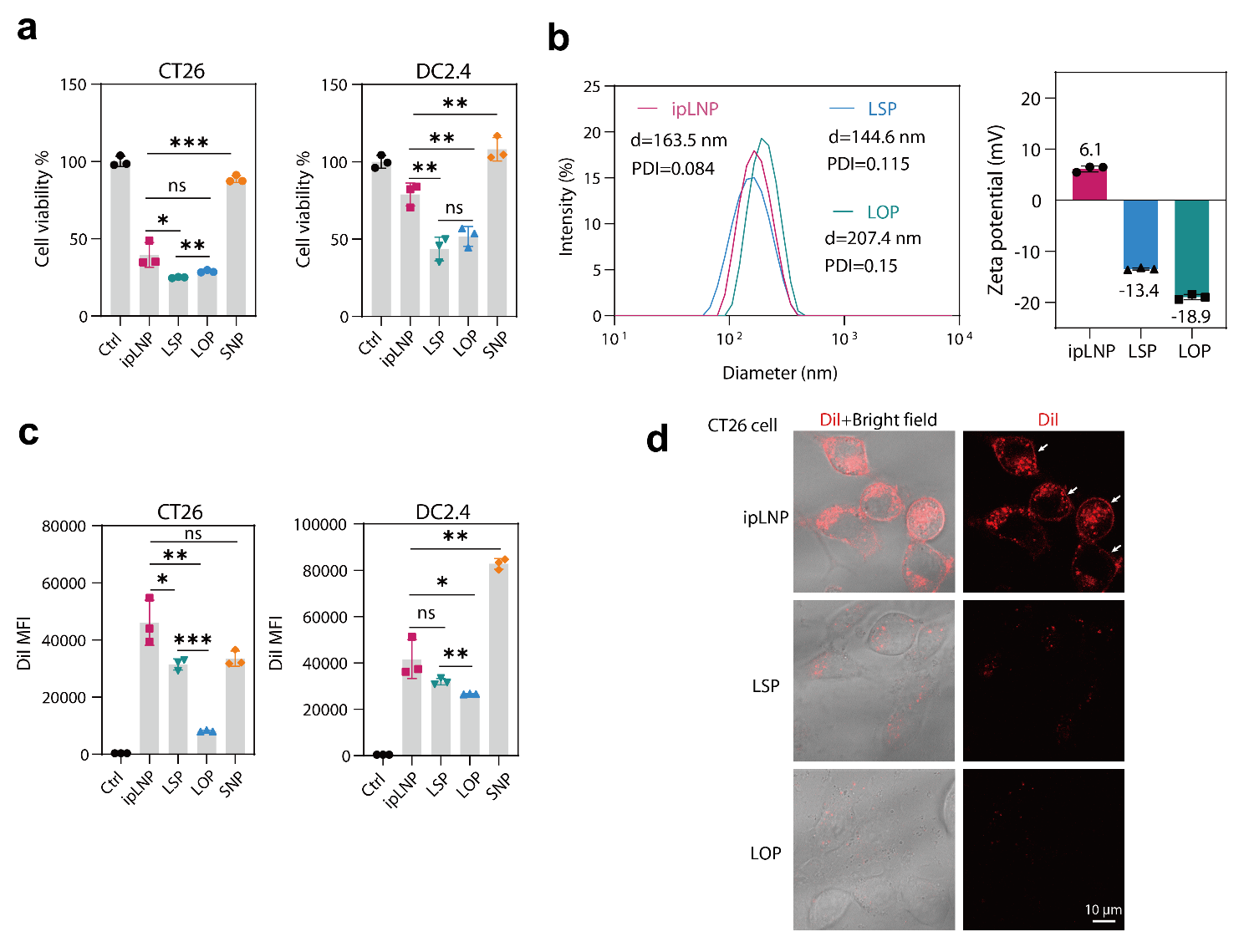


**Figure S4.** **a**, Cell viability of CT26 and DC2.4 treated with ipLNP, LSP, LOP and SNP (IP9: 6 μg/mL, SM-102: 7.2 μg/mL) for 9.5 h and analyzed by CCK8. **b**, Size and zeta potential of ipLNP, LSP and LOP determined by Malvern Zetasizer Nano ZSE. **c**, Cellular uptake levels of CT26 and DC2.4 treated with DiI-labeled ipLNP, LSP, LOP and SNP (IP9: 5 μg/mL, SM-102: 7.2 μg/mL, molar ratio: IP9/DiI=11/1 and SM-102/DiI=11/1) for 3 h and analyzed by Agilent NovoCyte 3130. **d**, Confocal imaging of CT26 cells treated with DiI-labeled ipLNP, LSP, LOP for 2 h (IP9: 5 μg/mL, molar ratio: IP9/DiI=11/1) and analyzed by ZEISS LSM 780. ipLNP, IP9 : DDAB : Chol : DMG-PEG2000= 60 : 30 : 40 : 0.4. LSP, IP9 : DSPC : Chol : DMG-PEG2000= 50 : 10 : 38.5 : 1.5. LOP, IP9 : DOPE : Chol : DMG-PEG2000= 35 : 16 : 46.5 : 2.5. Data were shown as mean±SD and statistical significance was analyzed with two-tailed Student’s *t*-test, ns no significance, **p*<0.05, ***p*<0.01, ****p*<0.001.


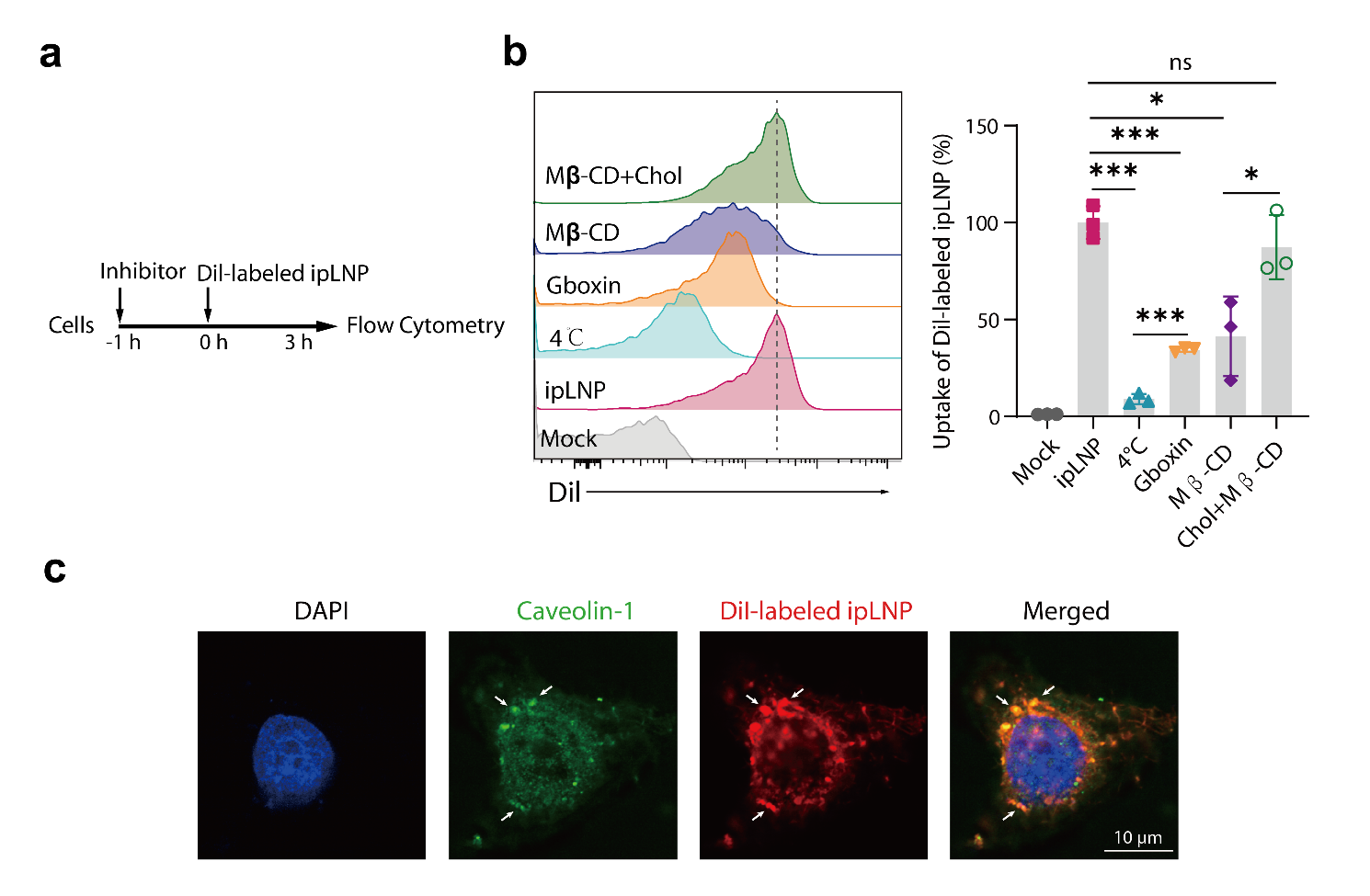


**Figure S5.** Cellular uptake mechanism analysis of ipLNP. **a**, Schematic of cells treated with inhibitors and ipLNP containing 4% DiI (molar ratio). **b**, Representative histograms and quantification of DC2.4 incubated with indicated conditions (IP9 5 μg/mL, oxidative phosphorylation inhibitor Gboxin 80 μM, lipid raft inhibitor Mβ-CD 2.5 mM, and cholesterol 120 μM). Chol+Mβ-CD: Mβ-CD was first mixed with cholesterol and further added into cells incubating for 1 h. **c**, Representative confocal images about the colocalization of ipLNP and caveolin in cells. DC2.4 cells were treated with ipLNP (IP9: 5 μg/mL) containing 4% DiI (molar ratio) for 3 h and then subjected to immunofluorescence sample preparation using caveolin-1 antibody (Proteintech). White arrow indicated the typical colocalization of ipLNP and caveolin. Data were shown as mean±SD and statistical significance was analyzed with two-tailed Student’s *t*-test, ns no significance, **p*<0.05, ****p*<0.001.


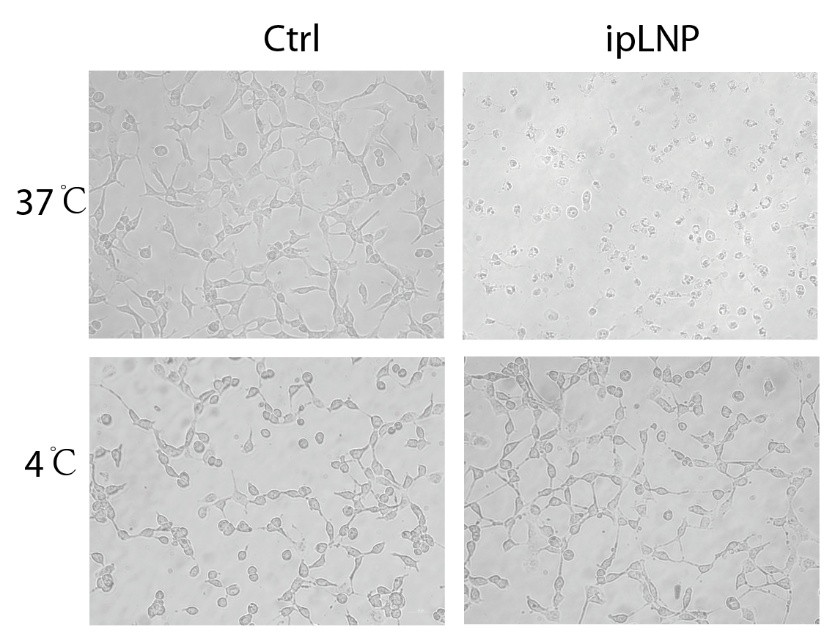


**Figure S6.** Representative images of CT26 cells treated with indicated conditions (IP9: 15 μg/mL).


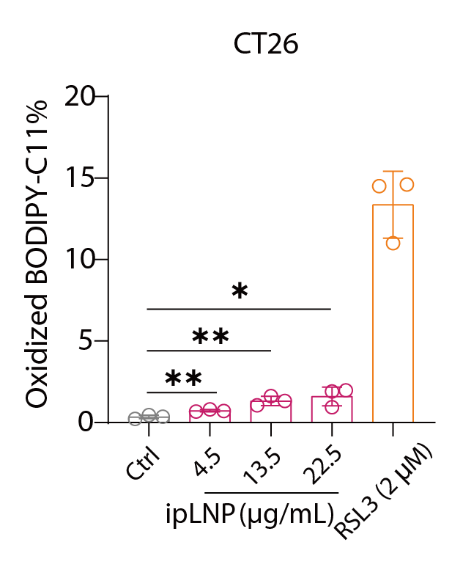


**Figure S7.** Lipid peroxidation analysis of CT26 cells using BODIPY-C11 probe. RSL3 was used as positive control. Data were shown as mean±SD and statistical significance was analyzed with two-tailed Student’s t-test, **p*<0.05, ***p*<0.01.


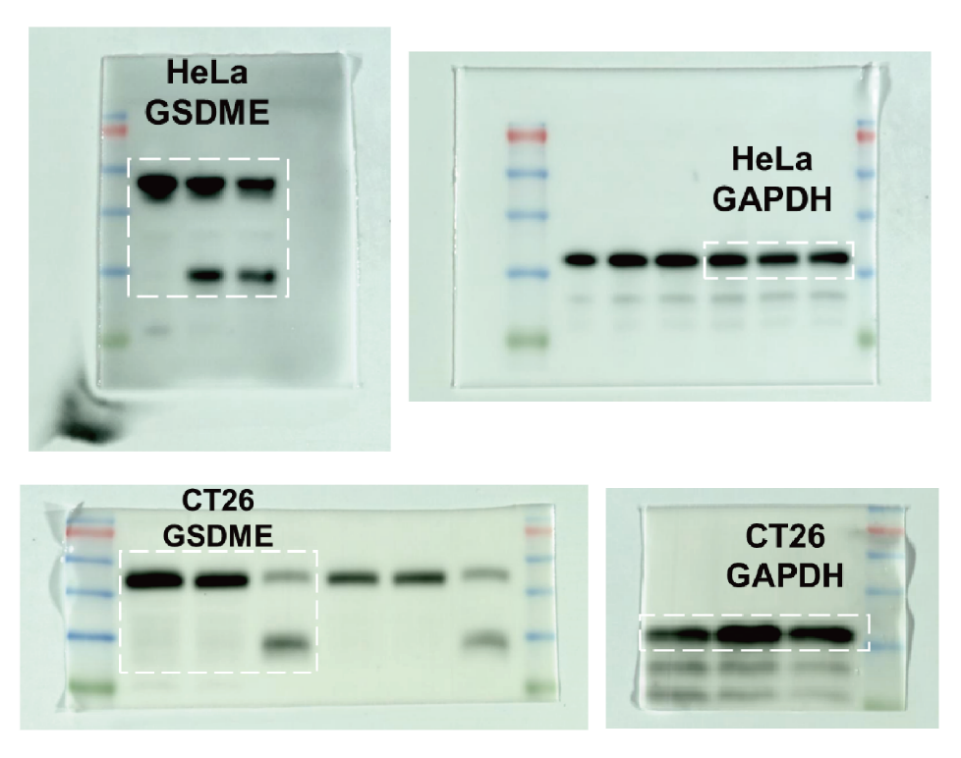


**Figure S8.** The original uncropped images of western blot for GSDME and GSDME NT analysis.


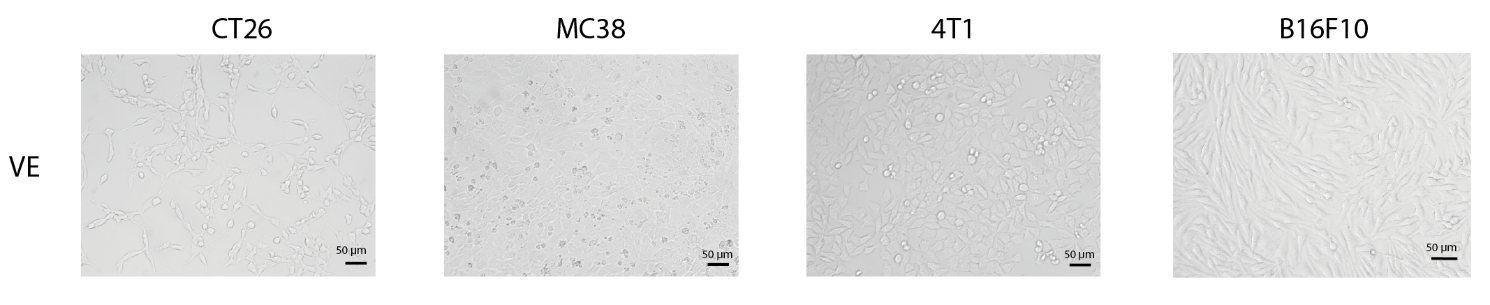


**Figure S9.** Representative images of indicated cells treated with VE.


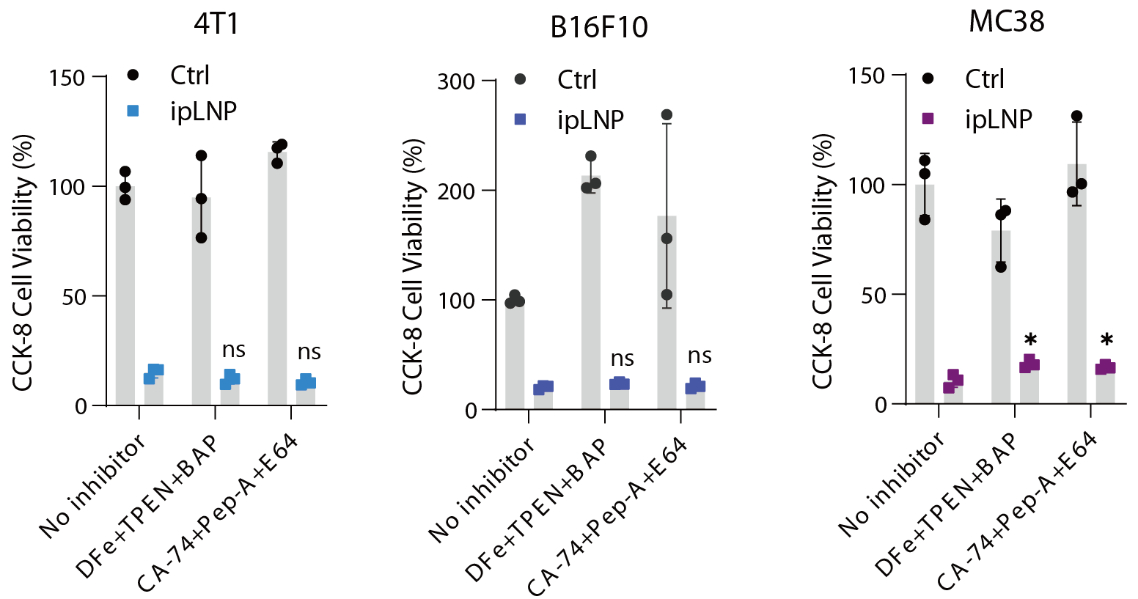


**Figure S10.** Cell viability of indicated cell lines treated with cathepsin inhibitors (CA-074+Pep-A+E-64) and metal chelators combination (DFe+TPEN+BAPTA-AM). IP9: 5 μg/mL for 4T1 and MC38, 6 μg/mL for B16F10. Data were shown as mean±SD and statistical significance was analyzed with two-tailed Student’s t-test, ns no significance, **p*<0.05.


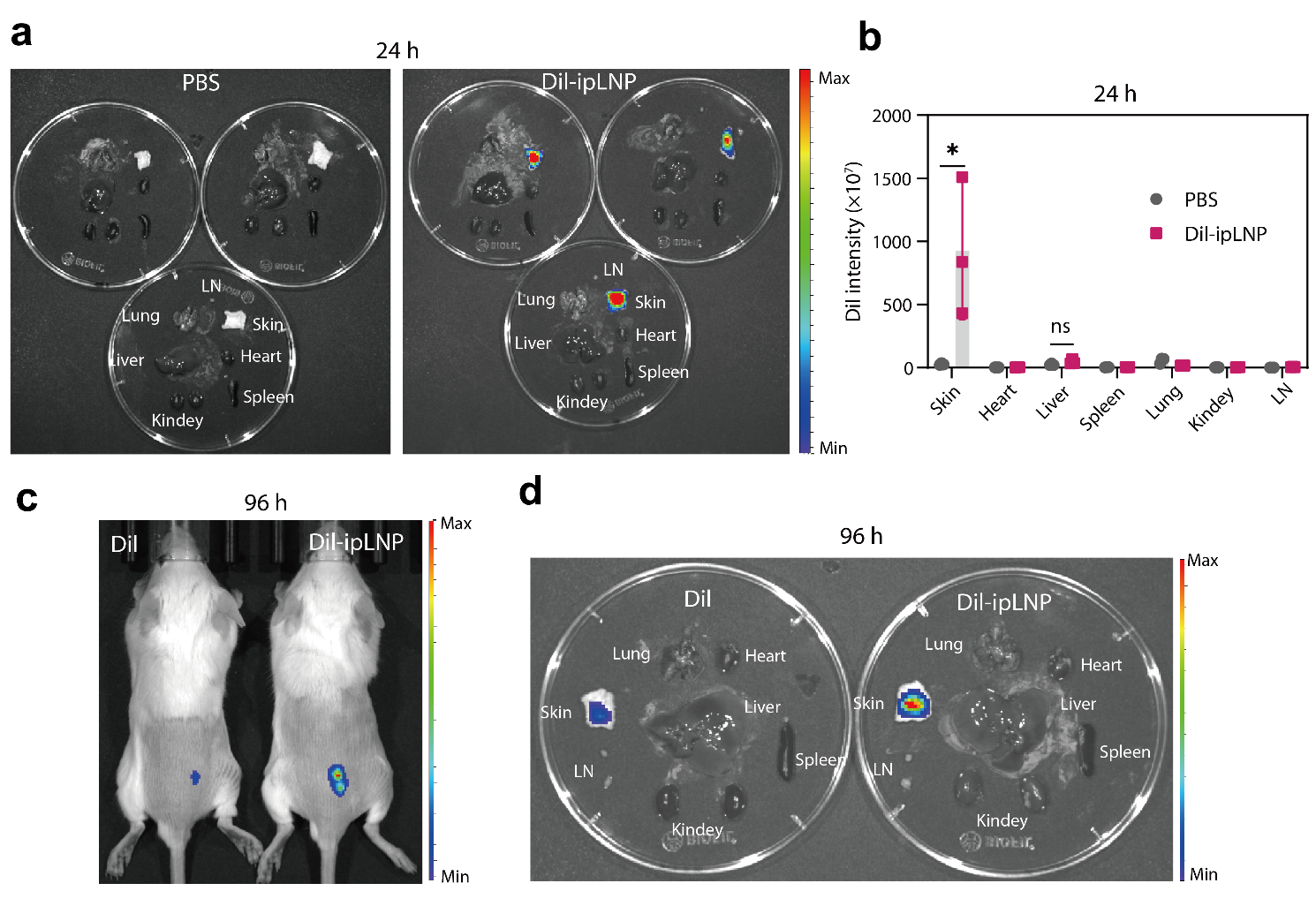


**Figure S11.** The tissue distribution of DiI-labeled ipLNP via intradermal injection. ipLNP containing 1% DiI (molar ratio) was intradermally injected into the right flank of BALB/c mice (IP9 dose: 90 μg) for fluorescence IVIS imaging and quantification (IVIS Spectrum, PerkinElmer). **a,b**, Fluorescence IVIS imaging and intensity quantification of skins at injected site, inguinal lymph nodes (LNs) and main organs at 24 h post injections. PBS was used as control. **c,d**, Fluorescence IVIS imaging of mice, skins at injected site, inguinal lymph nodes (LNs) and main organs at 96 h post injections. Free DiI was used as control. Data were shown as mean±SD and statistical significance was analyzed with two-tailed Student’s *t*-test, ns no significance, **p*<0.05.
